# Residual Strain and Nanostructural Effects during Drying of Nanocellulose/Clay Nanosheet Hybrids: Synchrotron X-ray Scattering Results

**DOI:** 10.1021/acsnano.3c03664

**Published:** 2023-08-02

**Authors:** Lengwan Li, Pan Chen, Lilian Medina, Lin Yang, Yoshiharu Nishiyama, Lars A. Berglund

**Affiliations:** † Department of Fibre and Polymer Technology, Wallenberg Wood Science Center, KTH Royal Institute of Technology, 10044 Stockholm, Sweden; ‡ Beijing Engineering Research Centre of Cellulose and Its Derivatives, School of Materials Science and Engineering, Beijing Institute of Technology, 100081 Beijing, People’s Republic of China; § NSLS-II, Brookhaven National Laboratory, Upton, New York 11973, United States; ∥ Univ. Grenoble Alpes, CNRS, CERMAV, 38000 Grenoble, France

**Keywords:** nanocomposite, layered silicate, biocomposite, orientation, Scherrer size

## Abstract

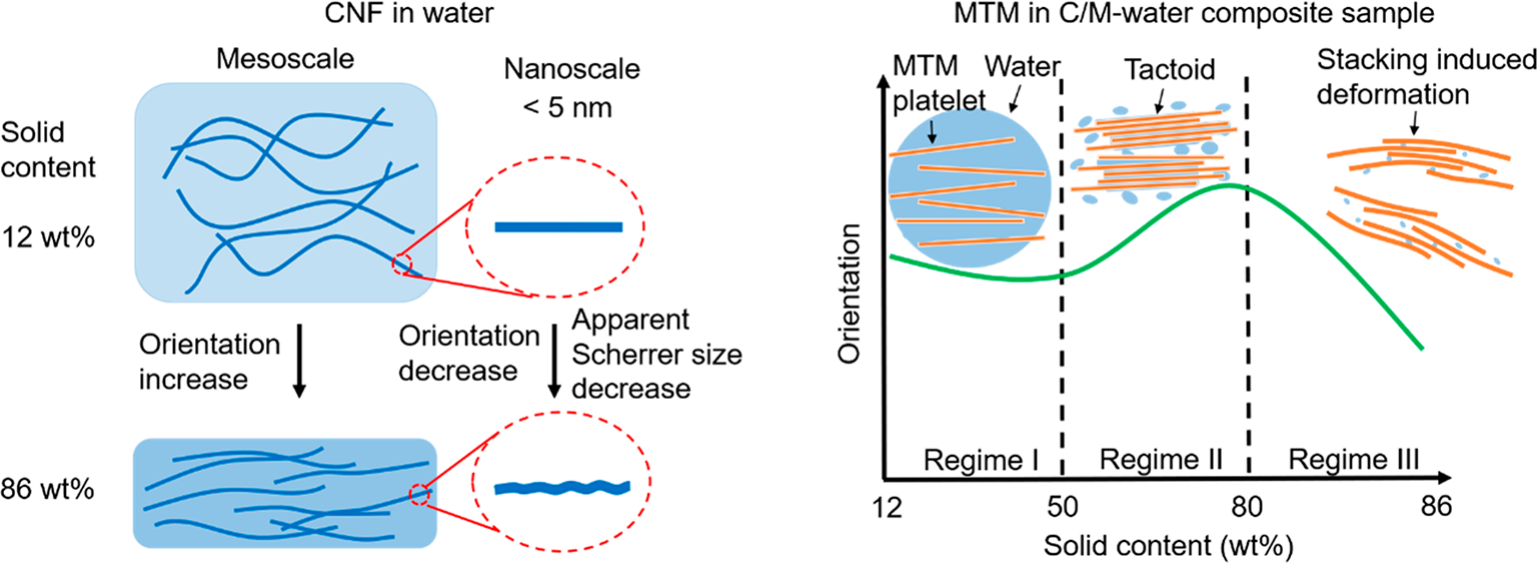

Cellulose nanofibrils (CNF) with 2D silicate nanoplatelet reinforcement readily form multifunctional composites by vacuum-assisted self-assembly from hydrocolloidal mixtures. The final nanostructure is formed during drying. The crystalline nature of CNF and montmorillonite (MTM) made it possible to use synchrotron X-ray scattering (WAXS, SAXS) to monitor structural development during drying from water and from ethanol. Nanostructural changes in the CNF and MTM crystals were investigated. Changes in the out-of-plane orientation of CNF and MTM were determined. Residual drying strains previously predicted from theory were confirmed in both cellulose and MTM platelets due to capillary forces. The formation of tactoid platelet stacks could be followed. We propose that after filtration, the constituent nanoparticles in the swollen, solid gel already have a “fixed” location, although self-assembly and ordering processes take place during drying.

## Introduction

Nanostructural changes and formation of residual strains during drying of cellulose nanofibril (CNF)–montmorillonite (MTM) clay nanoplatelet composites (CNF/MTM) are reported based on synchrotron X-ray scattering data. To the best of our knowledge, these are important experimental data for nanocellulosic materials showing residual drying strains at the nanoscale. This result, as well as clay agglomeration and CNF orientation effects during drying, has implications for the processing and materials design of CNF nanocomposite films.

CNFs are flexible, high-strength fibrils disintegrated from chemical wood pulp fibers.^1^ Typical diameters are in the 3–10 nm range, with lengths in the 0.7–2 μm range. Low-porosity (<20%, more commonly <10%) films can be prepared by filtration and drying of CNF hydrocolloids.^2^ Clay (e.g., MTM) nanoplatelets can be added to the colloid to form strong, transparent, fire-retardant nanocomposite gas barrier films with MTM nanoplatelets in a matrix of CNF.^3,4^ The use of oxidized CNF is important, since CNF dispersion and optical transmittance are improved.^4^ Water vapor resistance has been examined,^5^ and there was an early review on the wider topic of nanofibril/inorganic hybrids.^6^ Our CNF/MTM film research progress has included fire retardancy mechanisms,^7^ strong heat shielding function^8^ and the use of an epoxy matrix^9^ to limit moisture sensitivity. Xu et al. analyzed the importance of nanoparticle surface charge effects.^10^ Layer-by-layer assembly of CNF/2D nanosheets^11^ can provide controlled constituent layer thickness but is a much slower process than filtration. It was used in the pioneering polymer/clay nacre-mimetic composites study,^12^ and desirable nacre-like structures have also been used for electromagnetic interference shielding with MXene nanoplatelets.^13^ Nanoplatelets ordered in-plane in a polymer matrix in a nacre-like organization can provide very high reinforcement efficiency.^14^ In addition, the arrangement of nanoplatelets creates a tortuous path of gas–liquid diffusion, which improves gas–liquid barrier properties. In addition to the aforementioned references, Batchelor et al. developed CNF/MTM composites with very low water vapor permeability through vacuum filtration^15^ and spray coating processes.^16^ For industrial scaling of the present CNF/MTM nanocomposites, fast vacuum-assisted filtration should be combined with a high degree of ordering and dispersion of individual nanoplatelets; therefore, we must further investigate nanostructure formation during processing. Recently, nanostructural characterization by X-ray scattering supported efforts to improve the dispersion of nanoparticles for improved mechanical properties of CNF/MTM,^17^ also at high MTM clay content.

After filtration of the colloidal CNF/MTM mixture, the dry content of the wet cake is around 20–30%. During drying of the cake, structural changes are induced, but little is known about nanostructure formation in this step. The liquid medium is evaporated, and the organization of CNF fibrils as well as MTM clay platelets is adjusted and reaches low porosity due to capillary effects. Nanostructural details are important, since lower nanoporosity, higher orientation of fibrils,^18^ smaller out-of-plane MTM misalignment, and smaller MTM tactoid (aggregate) size^17,19^ will improve mechanical properties.

There has been little previous work on nanostructural analysis during drying of CNF composites, even for drying of neat CNF fibrils from the wet state. Nogi et al. investigated drying mechanisms in neat CNF films, but without X-ray diffraction, focusing on the reduction of nanoscale void content.^20^ They also showed that ethanol drying (also investigated here) results in nanopaper films of high haze (transmitted but scattered light) due to higher porosity.^21^ Newman et al. and Iversen et al. used ^13^C nuclear magnetic resonance (NMR) to investigate cellulose fibril aggregate dimensions during drying.^22,23^ Pore size and size distribution in cellulosic wood pulp fibers during drying have been examined by ^1^H and ^2^H NMR relaxation,^24^ differential scanning calorimetry (DSC),^25^ and scanning electron microscopy (SEM) or N_2_ sorption analytical tools.^26^ Li et al. investigated the macro- and microstructural changes in cellulose beads during drying from water and ethanol, using optical microscopy combined with *in situ* small-angle X-ray scattering (SAXS) and wide-angle X-ray scattering (WAXS) measurements.^27^

In previous work on CNF/MTM films, we reported on MTM tactoid size^17,19^ and CNF and MTM out-of-plane misalignment by XRD and related these compositional effects to mechanical properties.^17,18^ There seems, however, to be only one previous investigation of drying processes in CNF/2D platelet composites. Munier et al. used time-resolved SAXS to investigate structural changes in CNC/MTM composite droplets during drying.^28^ In a study of graphene oxide (GO) platelet–polymer matrix nanocomposites, Brinson and co-workers, however, investigated the formation mechanisms of PVA/GO and PMMA/GO^29^ and characterized interplatelet distance using XRD. They also studied formation mechanisms in PEO/GO nanocomposites processed by vacuum-assisted filtration, using XRD.^30^ Brinson’s lab also used flash-freezing as a function of time, to learn about formation mechanisms of vacuum filtered and dried GO papers.^31^

The most important previous study, inspiring the present work, was by Ogawa et al., who used molecular dynamics simulations.^32^ During drying of cellulose rods (cellulose nanocrystals), water evaporation and associated capillary forces induced substantial residual strains in the nanocellulose crystals.^32^ An experimental verification of this phenomenon is an important motive for the present study. It is focused on CNF/2D platelets, the use of synchrotron X-ray, and an emphasis on nanostructure formation and characterization. CNF as a semicrystalline matrix phase offers the possibility to follow the ordering of the matrix phase during drying. Our focus is on the drying phase after filtration, where nanoparticle aggregation takes place due to lateral capillary forces.^33^ We prepared CNF and clay colloids, and during the first filtration stage, wet cakes of CNF/MTM were formed. The micro-/nanostructural evolution of this cake during drying was followed by time-/space-resolved small-/wide-angle X-ray scattering (SAXS/WAXS). Lateral capillary forces strongly influence the “self-assembly” process. The changes in cellulose and MTM crystal strains and orientation were analyzed. An analysis technique for CNF/MTM nanocomposites was developed where the apparent Scherrer size and out-of-plane orientation index were estimated for both nanoparticles using 2D fitting of CNF and MTM patterns in the composite. Drying was carried out from water and ethanol to investigate effects from the corresponding differences in capillary forces.

## Results and Discussion

### Sample Preparation and X-ray Measurements during Drying

**Figure 1:**
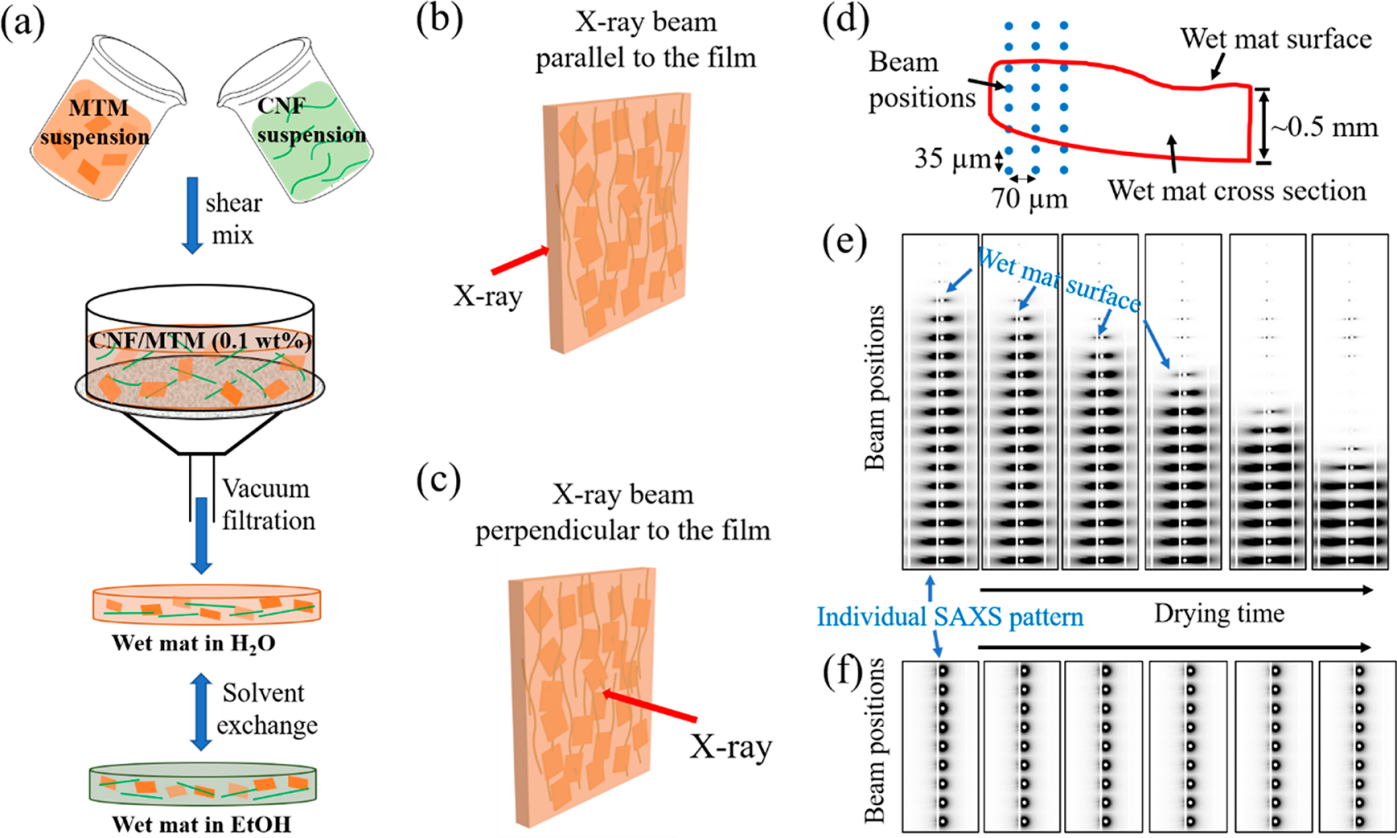
(a) Preparation of wet mats of CNF/MTM. X-ray scattering measurements with an incident X-ray beam (b) parallel or (c) perpendicular to the wet mats. (d) Schematic illustration of scanning X-ray with beam parallel to the wet mat surface. Examples of (e) anisotropic and (f) isotropic SAXS detector images.

Three types of wet cakes (mats) were prepared by vacuum filtration according to Figure 1: (1) neat CNF in water (CNF-water), (2) CNF/50 wt % MTM mixture in water (C/M-water), and (3) CNF/50 wt % MTM mixture in ethanol (C/M-EtOH). Figure 1a illustrates the preparation of the wet composite mats. The aim is to obtain nanostructured composites with discrete reinforcing MTM nanoplatelets in a continuous CNF matrix. A colloidal MTM dispersion in water was obtained by several cycles of centrifugation/sonication and then slowly added to a CNF dispersion to prepare well-dispersed colloidal mixtures of CNF and MTM. The dry content ratio of CNF and MTM was controlled as 50/50 wt %. The mixed CNF-MTM dispersion was then vacuum filtered to form a wet cake (C/M-water). C/M-EtOH was obtained by solvent exchange of the C/M-water mat.

**Figure 2:**
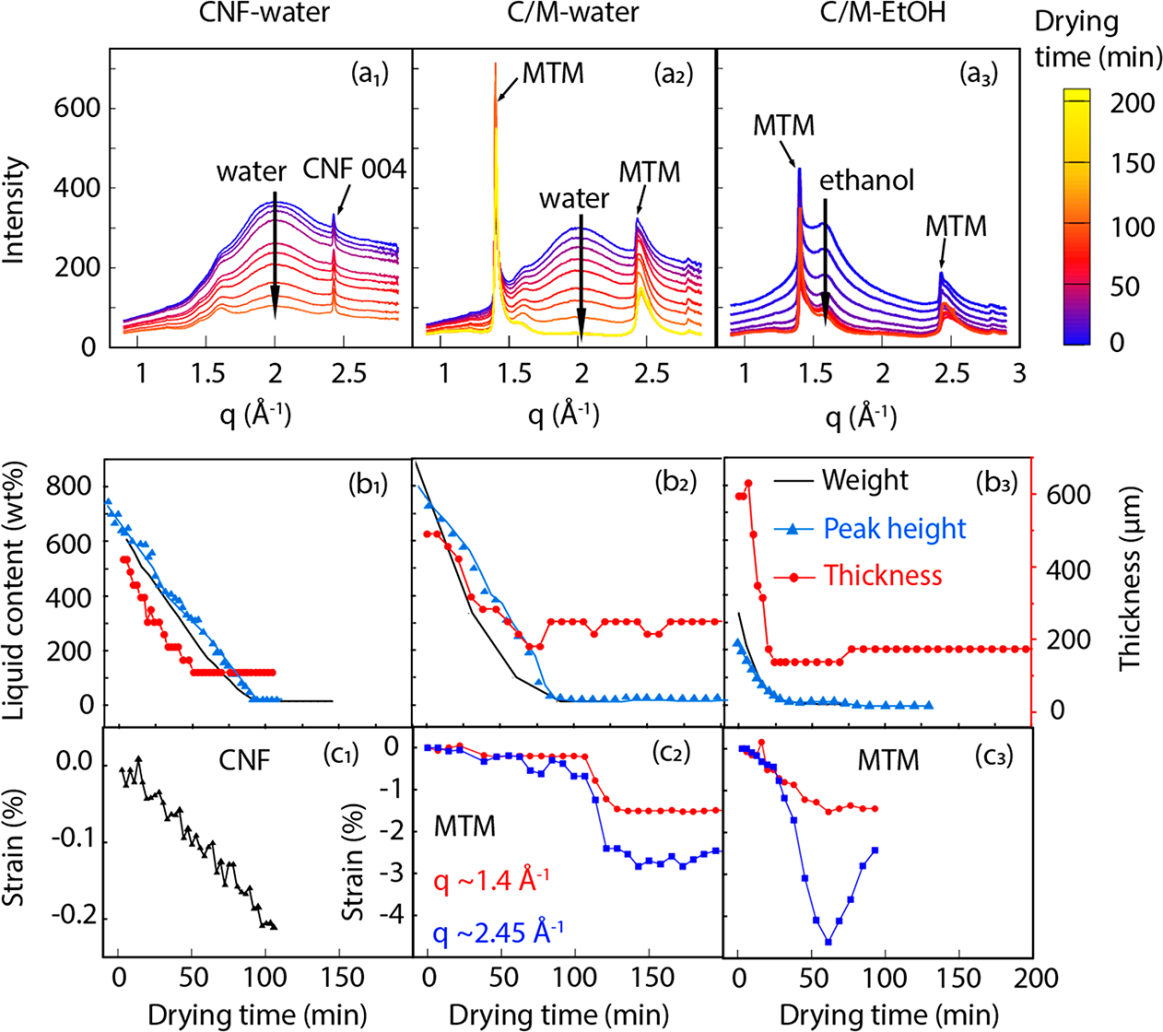
Intensity vs *q* curves of (a_1_) CNF-water, (a_2_) C/M-water, and (a_3_) C/M-EtOH wet cakes in the *q* region from 0.9 to 2.9 Å^–1^. The color bar indicates drying time. Liquid contents vs drying time of (b_1_) CNF-water, (b_2_) C/M-water, and (b_3_) C/M-EtOH determined by three different methods. Thickness is measured by film thickness in Figure 1. Strain (displacement ratio) vs drying time of (c_1_) CNF 004 in CNF-water (axial), (c_2_) MTM 110 and 200/130 (in-plane) in C/M-water, and (c_3_) MTM 110 and 200/130 (in-plane) in C/M-EtOH. The standard deviations of the parameters are smaller than 1%.

During drying, the wet mats were scanned by the X-ray beam parallel (Figure 1b) and perpendicular (Figure 1c) to the film surface. When probed with the X-ray beam perpendicular to the film surface (Figure 1c), isotropic patterns were obtained at all positions and the pattern did not vary with position (Figure 1f). Thus, the structure is random-in-the-plane when averaged over the sample volume in the beam, i.e. 5 μm length scale, and is also homogeneous at the scale of the whole sample. The central scattering of this scan as a function of time was aligned to show the overall data feature as shown in Videos S1–S3. For the “parallel” case (Figure 1d), each line scan contains 20 exposures which are 35 μm apart in the thickness direction (vertical in Figure 1e) and 70 μm apart in the horizontal direction. The line scan interval depends on the drying kinetics of different material compositions: ∼2 min for CNF-water and C/M-EtOH samples, ∼7 min for C/M-water sample. Figure 1e illustrates some examples of SAXS data with the X-ray beam parallel to the sheet surface. Positions with no scattering patterns are because of sample shrinkage. We can then determine the change of film surface position as a function of shrinkage and also wet mat thickness change as a function of time (Figure 2b). During drying, the initial thickness ranges of CNF-water, C/M-water, and C/M-EtOH wet mats are about 400–100, 500–220 and 600–180 μm, respectively.

The drying rate under ambient conditions was measured separately by simple weighing (Figure 2b). The liquid content is defined as the liquid content/dry content. The decrease is linear with time, meaning a constant evaporation rate. The moisture content stabilized in the range of ∼10–15% at the end. The evaporation rate is slow for C/M-water composites and higher for C/M-EtOH wet composites. We estimated the liquid evaporation coefficient (θ, kg m^–^^2^ h^–1^) as shown in Table S1. Free water surfaces show a value of 25 kg m^–2^ h^–1^, whereas neat CNF, C/M-water, and C/M-EtOH have values of ∼12.32, 3.62, and 4.37 kg m^–2^ h^–1^, respectively. The data illustrate the hindering effect of MTM nanoplatelets on evaporation rate. The path length for flow or diffusion of liquid molecules is strongly increased, since the present MTM platelets typically have thicknesses of 1 nm with average “diameters” of around 100 nm.^19^

### Crystal Strain of MTM during Drying (X-ray Perpendicular to the Film)

In this section, we discuss the data in Figure 2. Intensity versus *q* curves are presented (Figure 2a) as well as data for liquid content (Figure 2b) and residual strains in CNF and MTM (Figure 2c), all as a function of drying time.

The liquid content could be estimated for the case of the incident X-ray beam being perpendicular to the film. The isotropic scattering patterns (Figure 1f, Figure S2, and Videos S1–S3) were azimuthally averaged to one-dimensional intensity profiles as a function of the scattering vector *q* (Figure 2a and Figure S3). The change in the water or ethanol content in the sample was estimated from the intensity of the broad peak at *q* = 2 Å^–1^ (water) or *q* = 1.6 Å^–1^ (ethanol). Data correlated well with the liquid content estimated from the weight measurements (Figure 2b, in the second row).

During drying, CNF and MTM nanoparticles are brought in close contact with each other (CNF-CNF, CNF-MTM, or MTM-MTM contacts) due to capillary forces.^17^ Based on MD simulations,^32^ one may suspect that CNF fibrils could be subjected to substantial local deformation from capillary forces. In the CNF/water system, the diffraction from cellulose is almost constant. However, peak fitting of the 004 reflection at *q* = 2.42 Å^–1^ reveals a steady decrease in *d* spacing with drying time, with a final axial in-plane strain in CNF fibrils of about −0.2% (see Figure 2c_1_). This result is very important, as it demonstrates considerable residual drying strains in the CNF.

The mechanics problem causing this effect needs some discussion for a CNF film with a random-in-the-plane CNF orientation. The in-plane shrinkage was very limited during the drying experiment. In a particular reference direction, domains of transversely oriented CNFs would like to shrink during evaporation, although the very stiff axially oriented CNFs counteract this shrinkage since there is strong interaction between CNFs. As a consequence, those axial fibrils become subjected to axial in-plane compression during drying, and crystal strains are increasing almost linearly with time (Figure 2c_1_). If the axial CNF modulus is around 100 GPa,^34^ a compressive strain of 0.2% in the cellulose crystal results in a stress of 200 MPa.

In the CNF/MTM mixture, there are relatively sharp peaks at the positions *q* ≈ 1.4 Å^–1^ and *q* ≈ 2.45 Å^–1^ (Figure 2a and Figure S4) from MTM platelets, which are absent in samples without MTM.^35^ At the start of drying, the peak position was at *q* = 1.4 Å^–1^, corresponding to an in-plane *d* spacing of 4.49 Å, which is close to the theoretical value of *d*_110_. Similarly, the peak position of *q* = 2.45 Å^–1^ was close to the theoretical value of *d* = 2.433/2.430 Å^–1^ of the 200/130 reflection. Further details with respect to interpretation of peaks in Figure 2a are provided in the Supporting Information.

Data for MTM platelet crystal *d* spacing are important. Both MTM peaks (1.4 and 2.45 Å) show remarkable changes at the final stage of drying when the scattering peak from the liquid disappears. In C/M-water, both peaks are shifted to higher angle (Figure S4), and the corresponding in-plane *d* spacing dropped by about 1%. This suggests isotropic in-plane compression of MTM clay platelets by 1%, at the final stage (see Figure 2c_2_). A crystal strain of 1% seems excessive; this phenomenon is instead related to the formation of tactoid of platelets stacked on top of each other, resulting in a significant modification of the MTM unit cell dimension compared with the single platelet, which we call “stacking-induced deformation”. In a single platelet, the crystal is essentially 2-dimensional, but as another platelet is added to form a tactoid, the crystal dimensions are distorted. The phenomenon is present also for C/M-EtOH. It showed an even larger drop in MTM *d* spacing of 200/130 by as much as −4.5% (Figure 2c_3_), since the tendency for MTM tactoid formation was stronger for EtOH drying, as indicated in Scherrer Size of CNF and MTM during Drying.

Strong support for stacking-induced deformation is obtained from XRD data with the beam parallel to the film surface (see the next section), where Figure S6 in the Supporting Information provides evidence (reduced 1D WAXS pattern). In the C/M-water wet cake state, MTM platelets are individualized and there is no 001 peak for MTM. As drying proceeds, the 001 peak related to the MTM crystal appears, which means we have tactoid formation since 001 cannot be present without stacking of MTM platelets. Finally, Figure 2c_3_ shows that after drying of C/M-EtOH under ambient conditions for 60 min, the *d* spacing of 200/130 increased to values similar to those in C/M-water, suggesting some moisture-induced swelling.

### Analysis of Anisotropic Patterns (X-ray Beam Parallel to Film Surface)

**Figure 3:**
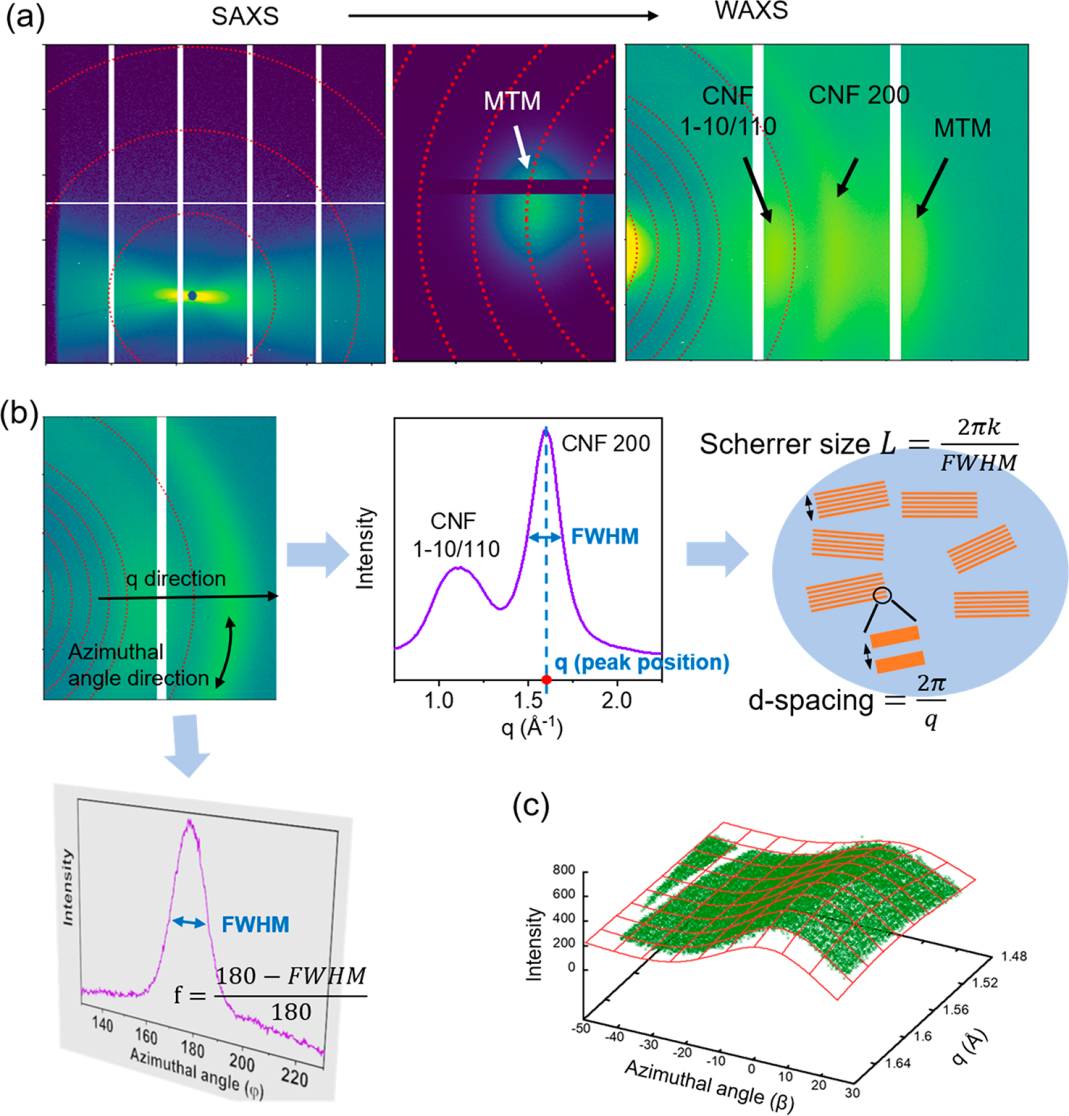
(a) Example X-ray patterns of a C/M-water sample obtained from a single exposure when the X-ray beam is parallel to the wet cake surface. Three individual patterns from left to right cover the *q* regions of 0.008–0.40, 0.3–1.4, and 0.5–2.8 Å^–1^, respectively. (b) Schematic diagram illustrating the degree of orientation, Scherrer size, and *d* spacing. The parameter fwhm is the full width at half-maximum of the peak, and *k* is the Scherrer constant. (c) One 2D fitting example of the CNF 200 pattern. The green dots are the data from experiment, while the red mesh is described by a 2D Gaussian equation.

In general, X-ray data from organic–inorganic hybrids composed of two different crystals could be very complex. Here, we are a bit lucky and can develop an analytical procedure to separate signals from the two constituents (CNF and MTM). When the X-ray beam is parallel to the mat surface (Figure 1b), anisotropic 2D patterns are observed (Figure S5 and Videos S1–S3). Figure 3a shows an example of X-ray scattering patterns on three different detectors captured from the C/M-water sample. The SAXS pattern shows oriented streaks, while the WAXS patterns show arcing. CNF (1–10/110) and (200) diffractions and MTM diffraction are indicated in the WAXS patterns, which show characteristics along both the *q* direction and azimuthal angle direction.

Since the 2D WAXS patterns are anisotropic, a conventional analysis method of azimuthally averaged 1D curve (*I* vs *q*) or *q*-averaged 1D curve (*I* vs azimuthal angle β) is not accurate. A 2D fitting method was developed. We first converted the patterns to data points containing values for intensity and scattering vector *q* and β (Figure S7) to obtain a detailed description of the anisotropic patterns.

We were then able to extract the following parameters from the CNF and MTM patterns (Figure 3b): (1) the integrated intensity of CNF and MTM peaks, which can describe the density or quantity of the particles, (2) the degree of orientation along the azimuthal angle direction to quantify the alignment of CNF and MTM, (3) the Scherrer size of CNF fibrils and MTM platelets to quantify through-thickness (lateral) MTM tactoid and CNF aggregate size, and (4) *d* spacing values for lateral crystallographic changes in CNF and MTM. These parameters are obtained by fitting the patterns by 2D Gaussian functions (Experimental Section and Figure S8–S12). This 2D fitting is very helpful, since parameters from both the *q* and β directions are extracted simultaneously (Figure 3c).

### Detection of Structural Changes in Thickness Direction during Drying

**Figure 4:**
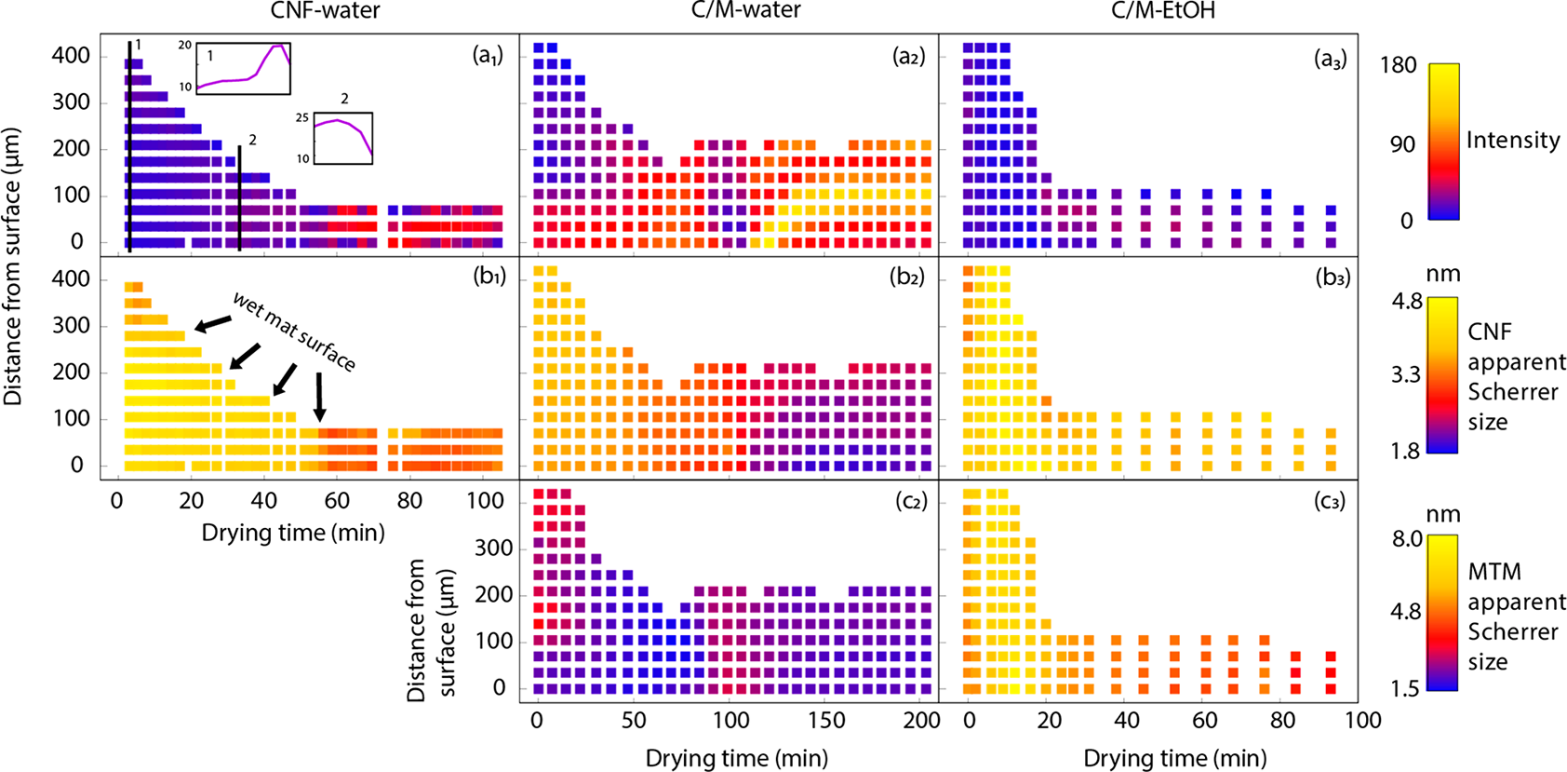
Palette images of peak intensity values integrated from CNF 200 patterns in (a_1_) CNF-water, (a_2_) C/M-water, and (a_3_) C/M-EtOH samples. Apparent Scherrer size obtained from CNF 200 patterns in (b_1_) CNF-water, (b_2_) C/M-water, and (b_3_) C/M-EtOH samples. Apparent Scherrer size of MTM (*q* close to 2 Å^–1^) in (c_2_) C/M-water and (c_3_) C/M-EtOH samples. In each image, the horizontal axis is drying time while the vertical axis indicates the distance of the X-ray beam center to one reference surface of the wet cakes, and the color bar represents the value of each parameter. Note that the interpretation of Scherrer size is discussed in Scherrer Size of CNF and MTM during Drying. The standard-deviations of the parameters are smaller than 1%.

We developed a methodology to detect intensity changes (related to density increase) and MTM and CNF peak broadening related to local Scherrer size during the drying process (see data in Figure 4). The integrated intensity of CNF and MTM peaks as a function of distance from surface and drying time is plotted in a color code (upper row of Figure 4). Note the reduced film thickness with time for all materials (fewer squares). The intensity is proportional to the quantity of particles interacting with the beam, and it increases consistently with drying time for all samples. As drying starts, the scattering contrast is between nanoparticles (CNF, MTM) and water. As the liquid content is reduced, drying involves moisture diffusion to a larger extent and the scattering contrast is between nanoparticles (CNF, MTM) and moist air, thus resulting in higher intensity. Composite samples (Figure 4a_2_,a_3_) show a trend similar to that for CNF-water.

Toward the end of the drying process, C/M-water shows higher intensity (more yellow and red) than C/M-EtOH due to lower porosity. The lower intensity for CNF-water is simply due to the lower CNF sample density. There was some heterogeneity observed in the beginning of the CNF-water experiment (Figure 4a_1_ and Figure S13), with one side of the sample showing a higher particle concentration. The sample experienced some drying before exposure, and there may be some through-thickness gradient in the liquid content. Also, in the vacuum filtration process, the vacuum side may have a higher particle concentration. This heterogeneity disappears during drying.

### Scherrer Size of CNF and MTM during Drying

We then progress to the interpretation of Scherrer size changes. This parameter is sometimes interpreted as being proportional to the nanoparticle size. For this drying process, it is instead strongly related to residual crystal strains from drying and peak broadening associated with the distribution of local drying strains. Figure 4b shows the through-thickness apparent Scherrer size values of the CNF 200 reflection during drying. All materials show a decreasing trend in Scherrer size. The reason is that during drying substantial lateral capillary effects are in operation. As the liquid is removed, it also pulls the nanoparticles together. This mechanism has been simulated at the molecular scale, and the cellulose crystal may become deformed, even plastically.^32^ Residual strains cause the apparent Scherrer size to be reduced, although there is also a contribution from peak broadening to this decrease. The C/M-EtOH sample (Figure 4b_3_) shows relatively larger through-thickness CNF Scherrer size than others (4.8–3.2 vs 4.0–1.8 nm). This is because the surface tension of ethanol is lower compared to that of water, so the CNF is subjected to lower capillary forces. The Scherrer size of MTM (lateral) in C/M-water (Figure 4c_2_) fluctuates with some increase at 100 min but shows a general decreasing trend in MTM Scherrer size from 4 to 1.5 nm (note that peak broadening is increasing the effect). This is related to shrinkage-induced deformation, as is supported by the corresponding decrease in the lateral MTM *d* spacings (Figure S14). The C/M-EtOH sample (Figure 4c_3_) shows a larger Scherrer size (8–3 nm).

### Orientation of CNF and MTM during Drying

**Figure 5:**
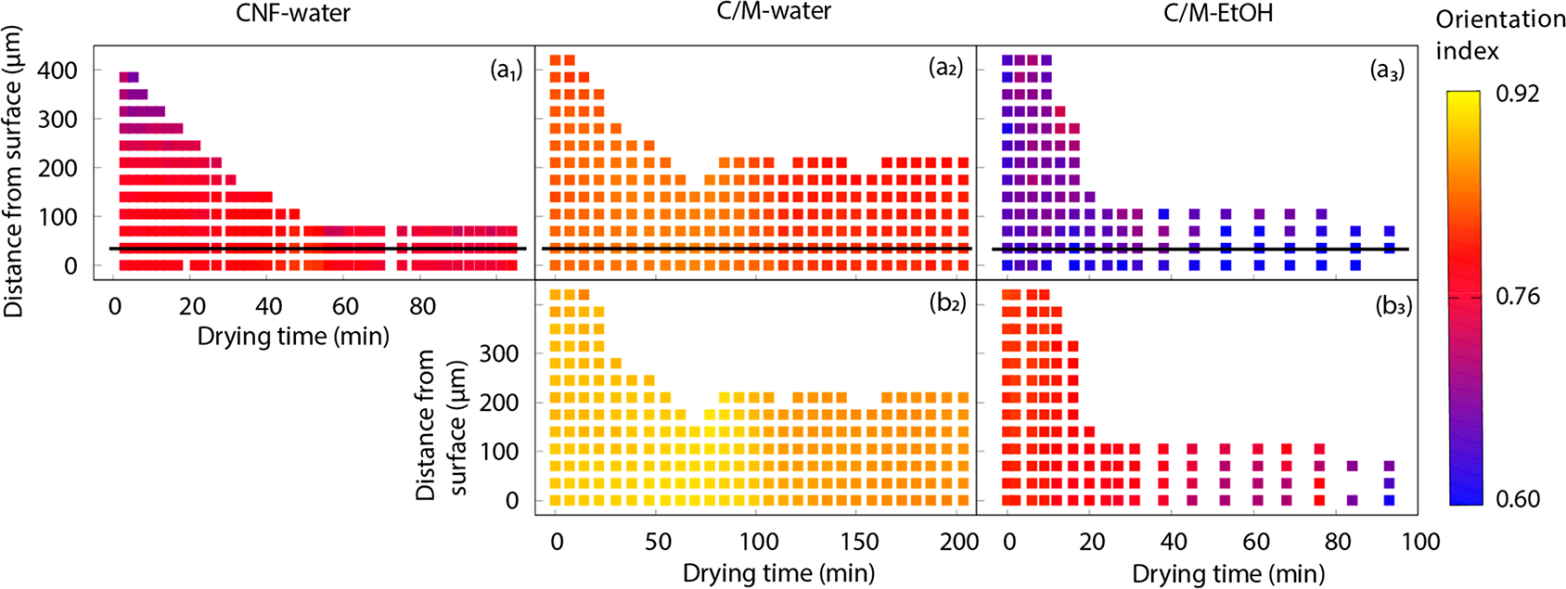
The first row shows the orientation indexes based on WAXS of (a_1_) CNF-water, (a_2_) C/M-water, and (a_3_) C/M-EtOH samples extracted from the CNF 200 pattern. The second row shows MTM orientation indexes of (b_2_) C/M-water and (b_3_) C/M-EtOH samples extracted from the MTM (*q* ≈ 2 Å^–1^) pattern. In each image, the horizontal axis is drying time while the vertical axis indicates the distance of the X-ray beam center to one reference surface of the wet cakes, and the color bar represents the value of each parameter. The standard deviations of the parameters are smaller than 3%.

Figure 5 shows the orientation index of CNF 200 and MTM (*q* ≈ 2 Å^–1^) obtained from WAXS. This orientation index of the CNF-water sample shows only small changes with an increased drying time. The data seem to state that the CNF in the C/M-water composite (Figure 5a_2_) has a stronger orientation than the CNF in the neat CNF-water (Figure 5a_1_). This is an artifact (explained in great detail in Figures S15 and S16 of the Supporting Information), and the real CNF orientation at WAXS scale is similar in the two systems.

In C/M-water, the orientation indices of both CNF (a_2_) and MTM (b_2_) are initially unchanged, followed by a trend of increased orientation. The reason for this increase is that the liquid content is lowered, so that capillary effects come into operation. Toward the end of the drying process, the local WAXS crystal orientation is again decreased since local deformation of MTMs (distortions due to neighboring particles) is taking place from capillary forces (Figure 5b_2_). Note that the overall CNF and MTM orientation indexes are higher for water than when drying from ethanol (Figure 5a_3_,b_3_).

**Figure 6:**
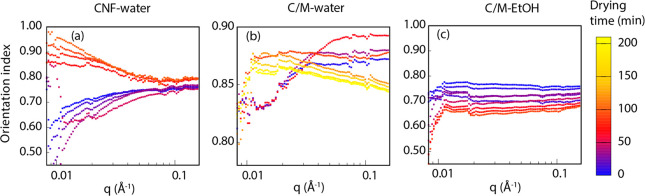
Orientation index as a function of *q* for different drying time. Anisotropic SAXS data for (a) CNF-water, (b) C/M-water, and (c) C/M-EtOH samples when the X-ray beam is parallel to the wet mat surface. The selected data points are indicated by the black line in Figure 5a. The colored bar represents the drying time. The low-*q* region corresponds to large length scale >30 nm.

The spatial organization of CNF and MTM at larger scale (>40 Å) is evaluated by SAXS, as a complement to the crystalline structures observed by WAXS. Anisotropic streaks were observed in the 2D SAXS patterns of CNF-water, C/M-water, and C/M-EtOH samples (Videos S1–S3). We decomposed them into 1D anisotropic (Figure 6) patterns and isotropic patterns (Figure S17) fitted to pseudo-Voigt functions (Experimental Section). The low-*q* region reflects the larger scale CNF scattering, while the high-*q* region represents the internal cellulose crystal structure. For water-drying, these two orientation indices show different values. The larger scale low-*q* region should be more relevant to the in-plane Young’s modulus of the films, since it is a global property.

During drying, both CNF-water and C/M-water show an increased orientation index in the low-*q* region so that the larger scale particle orientation is improved (Figure 6a,b). In the high-*q* region, CNF-water shows a relatively stable orientation of the internal crystal structure, with little change. This is similar to the trend obtained from WAXS. An interesting observation for C/M-water is that the high-*q* region (crystal structure) shows a decreased orientation index toward the end of the drying process. This is in support of the previously discussed data (*d* spacing, Scherrer size) and confirms residual deformation (e.g., local bending) in CNF after drying. In C/M-EtOH, capillary effects are insufficient, and the orientation index decreased in the overall *q* region during drying (Figure 6c). Results consistent with residual strain formation toward the end of drying was confirmed for C/M-EtOH in the high-*q* region. Figure 6a shows a jump in orientation between 50 and 60 min as quantified from the low-*q* region (>30 nm) scale. This is when the solid content reaches ∼80%. This suggests the start of strong capillary effects, and it correlates with the initial crystallographic deformation of CNF (see Figure 2c_1_).

### Nanostructural Changes during Drying

**Figure 7:**
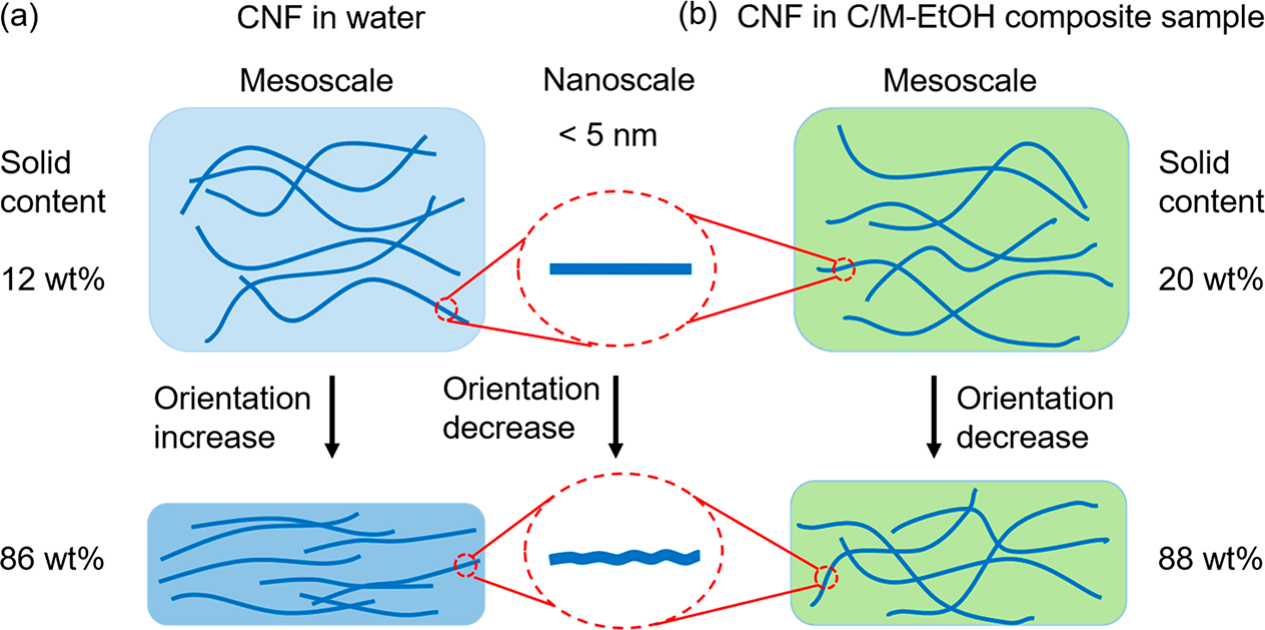
Schematic diagram illustrating the assembly of CNF fibrils during drying in water (CNF-water, C/M-water) and ethanol systems (C/M-EtOH). Note that the deformation of CNF fibrils is exaggerated.

**Figure 8:**
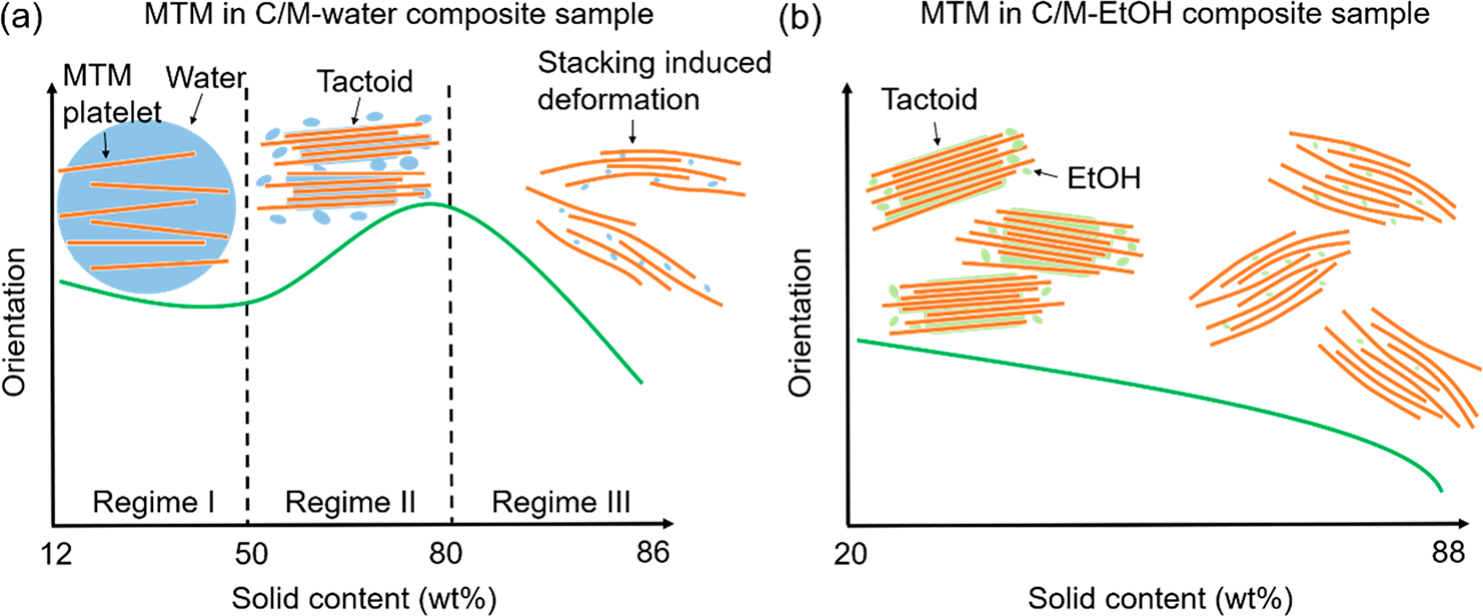
Scheme illustrating the nanoscale assembly of MTM platelets during drying in (a) C/M-water and (b) C/M-EtOH composite samples. Note that the stacking-induced deformation of MTM platelets is scaled up.

Based on the present results, the assembly process of CNF fibrils and MTM platelets during drying is illustrated (Figures 7 and 8).

(1) In the initial stage of drying from water, the solid content is only ∼12%. CNF in the CNF-water sample shows a Scherrer size of ∼4.6 nm with a surprisingly high orientation index of ∼0.84; In C/M-water, the CNF has a similar orientation index but smaller Scherrer size (∼4.0 nm) because well-dispersed MTM may decrease aggregation of CNF fibrils. As shown in Figure 7a, the mesoscale orientation of the fibrils is increased during drying, whereas the nanoscale orientation and apparent Scherrer size are decreased. Also, 200 *d* spacing in CNF from WAXS is changing (compression). The reason is that CNF nanoparticles are subjected to residual crystallographic deformation from capillary forces as nanoparticles are forced into intimate molecular contact. At the end of the drying process, the solid content is about 86 wt %. The discussion of CNF-EtOH in Figure 7b will follow in connection with Figure 8 (C/M-EtOH).

(2) During drying, the MTM assembly at the nanoscale is more complex and is separated into three regimes (Figure 8a). In the early stages of drying (Regime I), the apparent Scherrer size and orientation are relatively stable as the solid content is increased from ∼12 to ∼50 wt %; the platelets are already aggregated, but with high liquid water content in the galleries. In Regime II (∼50–80% solid content), MTM tactoids are formed and the MTM orientation and MTM Scherrer size are increasing. This regime is important since secondary interactions between nanoparticles are formed during this stage. When the solid content reaches 80 wt %, the MTM tactoids have a thickness of ∼4 nm (∼4 layers stacked together) and high orientation index (∼0.92). Toward the end stage of drying (Regime III, solid content from ∼80 to ∼86 wt %), MTM nanoparticles become subjected to residual crystallographic deformation with reduced Scherrer size (∼1.8 nm) and orientation values (∼0.80). The mesoscale MTM platelet orientation is increased in Regime II and then decreased in Regime III.

(3) After water/ethanol solvent exchange, the C/M-EtOH sample has an initial solid content of ∼20 wt %. Note that a large MTM tactoid is present at the very beginning of the C/M-EtOH wet cake (Figure S6d), which means the tactoid formation takes place during the solvent exchange. With further drying, the mesoscale orientation decreased (from ∼0.78 to ∼0.65) due to the rough surface (Figure S18) and high porosity (Table S2) of the film. On a crystal scale (Figure 8b), as a consequence of residual crystallographic deformation, the CNF fibrils and MTM platelets have lower orientation (∼0.6) and smaller apparent Scherrer size (∼4 nm).

In summary, the measured SAXS orientation (mesoscale) is increased in a water system, while WAXS orientation (crystal scale) decreases as CNF (and MTM) is subjected to significant residual deformation in the late stages of drying. For C/M-water, the nanoparticle orientation primarily improves during drying in Regime II (dry content from 50% to 80%) due to the formation of tactoids. For C/M-EtOH, the orientation on both meso-/nanoscale are decreased due to the rough surface, large porosity, and residual deformation. The presence of residual deformation in CNF should be a general behavior; when smaller molecules or colloids are in contact with the CNF network, this small molecule can spontaneously fill the voids,^36^ relaxing some of the residual strain. One may note that grazing incidence X-ray scattering studies can provide details about the nature of voids in the structure.^36^

We further investigated the mechanical properties of these samples after drying. As shown in Figure S19 and Table S2, addition of MTM increases the Young’s modulus. The CNF matrix is bonded to the MTM by substantial secondary bonds aiding stress transfer, and the structure is stratified and layered. The mechanism for the formation of these layers is not known in detail. Exchange of water to ethanol results in decreased mechanical properties of CNF films as well as of CNF/MTM composite films. This is due to the larger porosity, lower CNF and MTM orientation, formation of larger tactoids in the films, and higher surface roughness (Figure S18) when drying from ethanol. The larger scale low-*q* region orientation is the most relevant measure influencing the in-plane Young’s modulus of the films.

## Conclusions

Although vacuum-assisted self-assembly is a common processing route for 2D platelet nanocomposites, nanostructural development during solidification by drying is not well-known. This is also true for cellulose nanofibril (CNF) films and CNF/2D nanocomposites. The most important result from the present synchrotron X-ray diffraction investigation is that considerable residual strain is induced by drying, as confirmed in CNF and MTM crystals. Capillary forces are of critical importance in this context and for the self-assembly process. The random-in-space nature of the dilute hydrocolloidal CNF/MTM mixture is changed during filtration and drying into well-dispersed in-plane-oriented MTM platelets in a matrix of random-in-plane CNFs. The importance of capillary forces is supported by the difference in nanostructure between systems dried from high surface energy water and from lower surface energy ethanol (higher porosity, lower degree of in-plane CNF and MTM orientation, and larger MTM Scherrer (tactoid) size (7–8 layers).

The importance of drying shrinkage and associated CNF and MTM assembly should also be apparent; the present study shows the possibilities of measuring crystal scale deformation and orientation changes during drying solidification. After filtration, we suggest that constituent nanoparticles in the swollen gel have a fixed location, with respect to each other. During drying, self-assembly, ordering, and other interparticle interaction processes are taking place, which decide the final structural organization. The residual strain data collected here are important, since they confirm previous theoretical predictions. Residual drying strains will depend on drying conditions and influence global mechanical properties, the shape and geometry of the dried object, and the response to rehydration.

## Experimental Section

### CNF/MTM Wet Cake Preparation

The CNF suspension was prepared by an enzyme-assisted procedure, as described previously.^37^ In brief, pulp fibers (Nordic Paper AB) were washed and beaten, before an endoglucanase was added under slight heating, After mild enzymatic treatment, the suspensions were diluted and passed through a microfluidizer, resulting in an ∼1.37 wt % stable CNF suspension in water. The CNF is 6.6 ± 3.3 nm in diameter and ∼1 μm in length.^37^ MTM powder (CLOISITE-Na^+^, BYK Additives, Germany) was donated by Bjørn Thorsen AB. The MTM powder was shear mixed in deionized water with a percentage of 1 wt % by using an Ultra-Turrax shear mixer. The suspension was then subjected to several cycles of a sonication/centrifugation process until no sediments could be observed and collected in the bottom phase of the centrifugation tube. The stable upper supernatant was collected, and the solid concentration was determined to be ∼0.65 wt %. MTM suspensions were dropped into the CNF suspension under rigorous stirring by Ultra-Turrax, to form a cosuspension (0.1 wt %). The MTM was well-exfoliated into monolayers, with a lateral size of 131 ± 74 nm and thickness of ∼1.2 nm.^38^ The cosuspensions were subjected to vacuum filtration on a PVDF hydrophilic membrane with a pore size of 0.65 μm. The wet mats were peeled off from the membrane surface; some of them were further soaked in ethanol solvent to do solvent exchange. In the composite mats, the CNF and MTM have a dry content ratio of 50/50 wt %. The wet mats had a diameter of 85 mm, were sealed, and kept for further measurements.

### Small-Angle and Wide-Angle X-ray Scattering (SAXS and WAXS) Measurements

X-ray scattering measurements were carried out at the Life Science X-ray Scattering (LiX/ID16) of the National Synchrotron Light Source INSLS-II at Brookhaven National Laboratory, New York, US. Three Pilatus detectors, named SAXS, WAXS-1, and WAXS-2, were placed with sample-to-detector distances of 3.58, 0.71, and 0.34 m, respectively. The combination of the three detectors covers a scattering vector range of 3 > *q* > 0.005 Å^–1^, which was calibrated by using silver behenate. The SAXS detector covers the *q* range from 0.013 to 0.40 Å^–1^ and azimuthal angle range from −180 to 180°, while the WAXS-1 detector covers the *q* range from 0.5 to 2.8 Å^–1^ and azimuthal angle range from −40 to 60°, and the WAXS-2 detector covers the *q* range from 0.3 to 1.4 Å^–1^ and azimuthal angle range from 300 to 405°. For measuring with X-ray beam parallel to the sheet, the sheet was positioned vertically, and thus the WAXS detectors covered the reciprocal space roughly perpendicular to the sheet surface. Figure S1 shows a scheme of the sample loading.

### WAXS and SAXS Data Analysis

For each pixel, the corresponding azimuthal angle, β, and scattering vector amplitude, *q*, were calculated and stored in a file as an array. For each measurement, we get a series of (β_*i*_, *q*_*i*_*, I*_*i*_) sets with *i* = 1, ..., *n* where *n* is the total number of pixels. To obtain structural parameters from textured diffraction patterns, selected patterns in WAXS were fitted with a two-dimensional Gaussian function of β and *q*
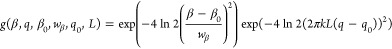
1where β_0_ and *q*_0_ are the peak positions along the azimuthal angle and *q* direction, respectively,*w*_β_ is the full width at half-maximum along the azimuthal direction, *L* is the Scherrer size,and *k* is a constant, taken here as 0.9. The baseline was assumed to be locally linear. The degree of orientation (*f*) is defined here as
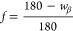
2

The SAXS patterns were decomposed into anisotropic and isotropic scattering intensity profiles. Pseudo-Voigt functions were used to fit the azimuthal intensity distribution profile at each *q*. The details were described by Nishiyama et al.^39^

## Supporting Information Available

Calculation method of liquid evaporation coefficient, explanation of the MTM XRD peaks in Figure 2a, descriptions of Videos S1–S3, scheme of the sample loading, examples of the 2D isotropic X-ray patterns, SAXS and WAXS curves in specific *q* regions when the X-ray beam is perpendicular to the wet mat surface, examples of the 2D anisotropic X-ray patterns, WAXS curves in specific *q* regions when the X-ray beam is parallel to the wet mat surface, X-ray intensity distribution as a function of scattering vector *q* and azimuthal angle (β), examples of the 2D fitting in specific *q* regions, palette image of peak intensity values integrated from the CNF 200 pattern in a CNF-water sample, palette images of MTM lateral *d* spacing values extracted from MTM, illustration of CNF planar orientation and MTM uniplanar orientation, experimental data and calculated data of CNF intensity profiles vs azimuthal angle, extracted isotropic SAXS curves when the X-ray beam is parallel to the wet mat surface, topographic features of membrane surfaces after drying, representative stress–strain curves of CNF and CNF/MTM samples, values of the variables and final evaporation coefficient calculation results, and mechanical property parameters of CNF and CNF/MTM samples (PDF)
Video S1: Overall X-ray scattering pattern feature vs drying time of CNF-water (MP4)
Video S2: Overall X-ray scattering pattern feature vs drying time of C/M-water (MP4)
Video S3: Overall X-ray scattering pattern feature vs drying time of C/M-EtOH (MP4)
