# Supplementary material for: Residual Strain and Nanostructural Effects during Drying of Nanocellulose/Clay Nanosheet Hybrids: Synchrotron X-ray Scattering Results
Source: ACS Nano. 2023 Aug 2;17(16):15810–20. doi: 10.1021/acsnano.3c03664 (PMC10448751; doi:10.1021/acsnano.3c03664)
Supplement: Supplementary file 1 — nn3c03664_si_001.pdf [file nn3c03664_si_001.pdf]

Supporting Information

Residual Strain and Nanostructural Effects during Drying of Nanocellulose/Clay Nanosheet Hybrids: Synchrotron X-ray Scattering Results

Lengwan Li [1], Pan Chen [1,2], Lilian Medina [1], Lin Yang [3], Yoshiharu Nishiyama [4,*],

Lars A. Berglund [1,*]

[1] Department of Fiber and Polymer Technology, Wallenberg Wood Science Center, KTH Royal Institute of Technology, 10044 Stockholm, Sweden

[2] Beijing Engineering Research Centre of Cellulose and Its Derivatives, School of Materials Science and Engineering, Beijing Institute of Technology, 100081 Beijing, P.R. China

[3] NSLS-II, Brookhaven National Laboratory, Upton, NY 11973, USA

[4] Univ. Grenoble Alpes, CNRS, CERMAV, 38000 Grenoble, France

Corresponding to:

Yoshiharu Nishiyama, Email: yoshiharu.nishiyama@cermav.cnrs.fr

Lars A. Berglund, Email: blund@kth.se

Supporting Text
Calculation of liquid evaporation coefficient of the wet mats

The liquid evaporation coefficient θ of the wet mats surface is described as:[1]

$$\theta = \frac{g_h}{A(x_s - x)} \tag{1}$$

where g_h is amount of evaporated water per hour (kg h^{-1}), A is water surface area (m^2), x_s is maximum humidity ratio of saturated air at the same temperature as the water surface (kg kg^{-1}) (kg H_2O in kg dry air), x is humidity ratio of the air (kg kg^{-1}) (kg H_2O in kg dry air).

$$x_s = 0.62198 p_w/(p_a - p_{ws}) \tag{2}$$

$$x = 0.62198 p_w/(p_a - p_w) \tag{3}$$

where p_{ws} is saturation pressure of water vapor, p_w is the partial pressure of water vapor in moist air, p_a is atmospheric pressure of moist air.

When the environment temperature is 25 °C, the parameters for the calculation and final θ values of the wet mats are listed in the Table S1. A and g_h are measured from the drying experiment directly. The p_a, p_w and p_{ws} values are obtained from literatures.[2, 3]

Table S1. Values of the variables and final evaporation coefficient calculation results θ. In the composites, the CNF and MTM have a dry content ratio of 50/50 wt %.

Samples	p_a (pa)	p_w (pa)	p_{ws} (pa)	A (m^2)	g_h (kg h^{-1})	θ (kg m^{-2} h^{-1})
neat CNF	101325	1565	3130	0.015386	0.001908	12.32
C/M-water	101325	1565	3130	0.007693	0.0002805	3.62
C/M-EtOH	101325	3934.5	7869	0.007693	0.0009166	4.37

When free water is evaporated, such as water evaporation from a swimming pool, the evaporation coefficient can be calculated as:[1]

$$\theta_{free} = 25 + 19v \tag{4}$$

v is the velocity of air above the water surface. In order to compare to drying of wet mats, we assume the velocity is 0, then θ_{free} has a value of 25 kg m^{-2} h^{-1}, which is in the same order of

the θ values of wet mats drying. However, the values of θ_{free} is larger than θ; this suggests that the CNF based wet mats have water retention properties.

Explanation of the MTM XRD peaks in Figure 2a

The intensity ratio of reflections 200:130 are reported to be of the order of 8:5, and the two contributions cannot be separated in the in-plane randomly oriented sample. Both peaks have asymmetric shape with tailing towards higher angle. This is related to the fact that the clay particle has highly anisotropic shape (individual crystal sheet is anisotropic due to the high aspect ratio, but in-plane orientation is random), and the diffraction spot is elongated in the reciprocal space perpendicular to the basal plane. Even when the center of the spot is not in the Bragg-condition, the tail of the spot overlaps the Ewald sphere giving rise to the intensity at higher angle.

Description of Video S1-S3

Video S1, S2 and S3 show the exposure patterns of CNF-water, C/M-water and C/M-EtOH samples, respectively. Each exposure pattern contains the scattering signals from CNF (MTM) nanoparticles and water (ethanol) with the detailed characteristics discussed in the main text. In the videos, each frame corresponding to one single line scan contains 20 exposures including SAXS (q range: 0.013-0.4 $Å^{-1}$) patterns on the upper row and WAXS (q range: 0.3-1.4 $Å^{-1}$ and 0.5-2.8 $Å^{-1}$) patterns on the two bottom rows. The left part on the videos shows isotropic patterns when X-ray beam is perpendicular to the wet mat surface, while the right part shows anisotropic patterns when X-ray beam is parallel to the wet mat surface. All the frames were aligned as a function of drying time to show the general geometry and liquid content evolution of wet mats.

The isotropic patterns primary shows the decreased intensity of liquid scattering as a function of drying time, the reduced 1D profiles were discussed in Figure 2 and S3-S4. The evolution of anisotropic patterns is more complex: With the increase of drying time, the CNF (MTM) scattering signals become more obvious while the water (ethanol) scattering intensity was reduced. Positions with no scattering patterns indicate shrinkage of the wet mat. The CNF-water and C/M-water samples showed lateral shrinkage with relatively uniform thickness in the end, while the C/M-EtOH sample showed thickness variation at the end stage of drying.

Supporting Figures

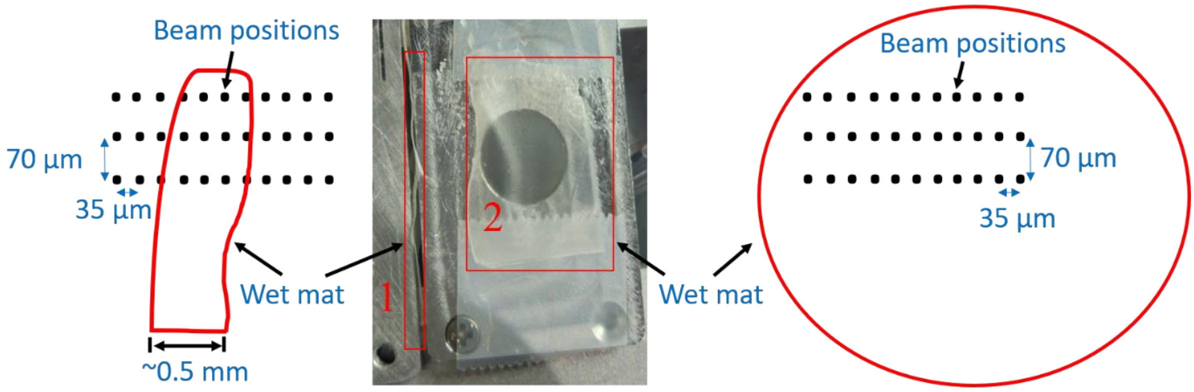

Figure S1. Wet mats were fastened at positions 1 and 2 of the sample stage at the beamline. The incident X-ray beam is perpendicular to the sample stage surface. At position 1, the beam is parallel to the film surface; at position 2, the beam is perpendicular to the film surface.

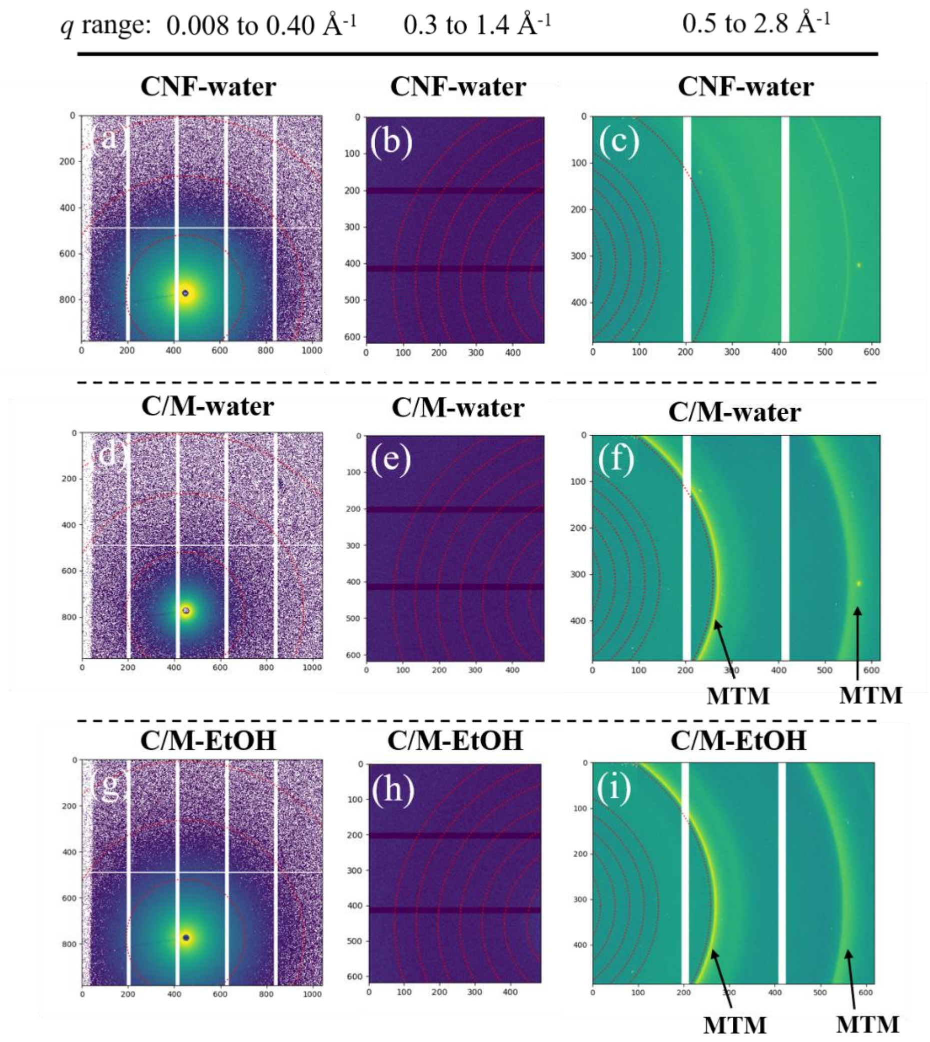

Figure S2. Examples of 2D X-ray patterns: (a, d, g) q region from 0.008 to 0.40 Å^{-1} (SAXS); (b, e, h) q region from 0.3 to 1.4 Å^{-1} (WAXS) and (c, f, i) q region from 0.5 to 2.8 Å^{-1} (WAXS) of CNF-water, C/M-water and C/M-EtOH samples. The X-ray beam is perpendicular to the film plane. Isotropic scattering is observed in both SAXS and WAXS patterns, and corresponds to random in-plane organization of CNF and MTM.

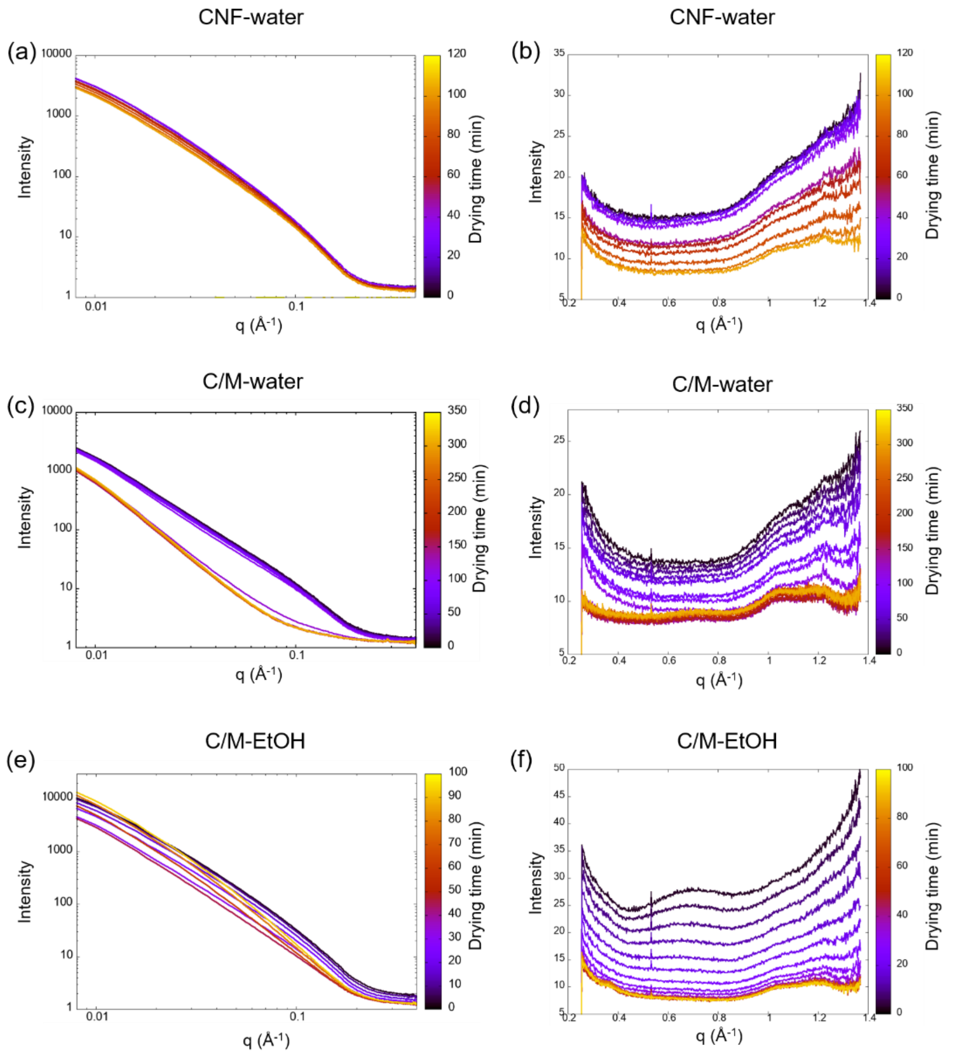

Figure S3. 1D SAXS curves (Intensity *vs.* q, q region from 0.08 to 0.40 Å^{-1}) of (a) CNF-water, (c) C/M-water and (e) C/M-EtOH during drying. 1D WAXS patterns (Intensity *vs.* q, q region from 0.3 to 1.4 Å^{-1}) of (b) CNF-water, (d) C/M-water and (f) C/M-EtOH during drying. The color bar indicates drying time. The X-ray beam is perpendicular to the wet mat surface.

Explanation of SAXS curves in Figure S3

With increased drying time, the CNF-water sample shows slightly decreased intensity, due to reduction of the film thickness. The C/M-water sample shows a bump in the beginning since the contrast is between water and particles; with further drying, the particles aggregated, and the bump disappeared. The C/M-EtOH sample has larger porosity compared to the C/M-water sample, and the larger contrast between CNF/MTM and air thus results in higher SAXS intensity.

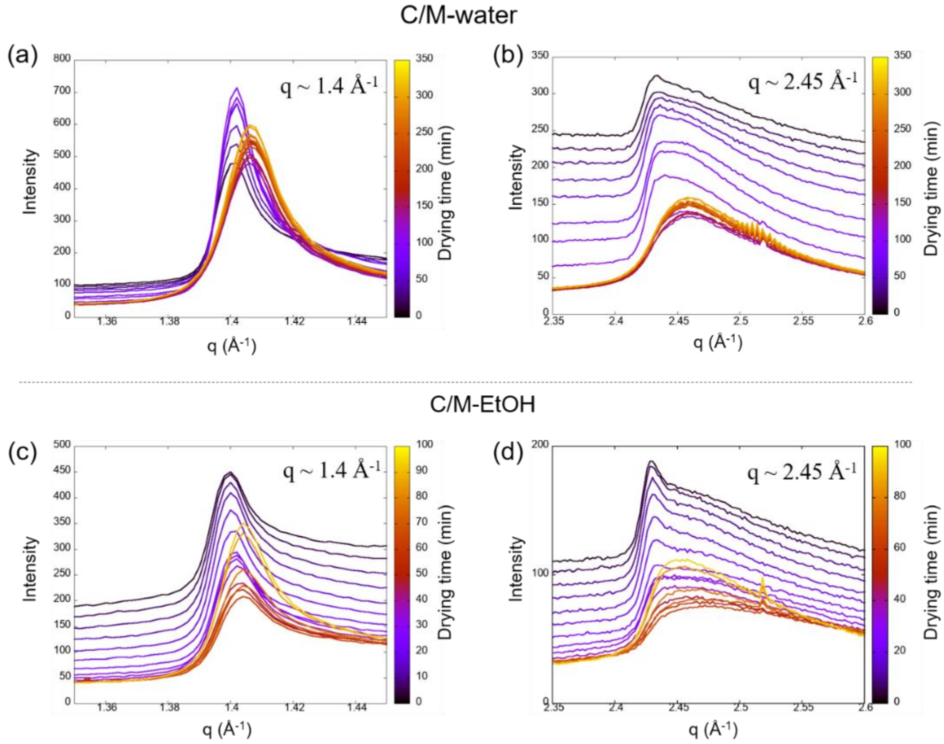

Figure S4. 1D WAXS curves (Intensity *vs.* q) with specific enlarged q regions (q ~ 1.4 Å^{-1} and q ~ 2.45 Å^{-1}) of (a, b) C/M-water and (c, d) C/M-EtOH during drying. The color bar indicates drying time.

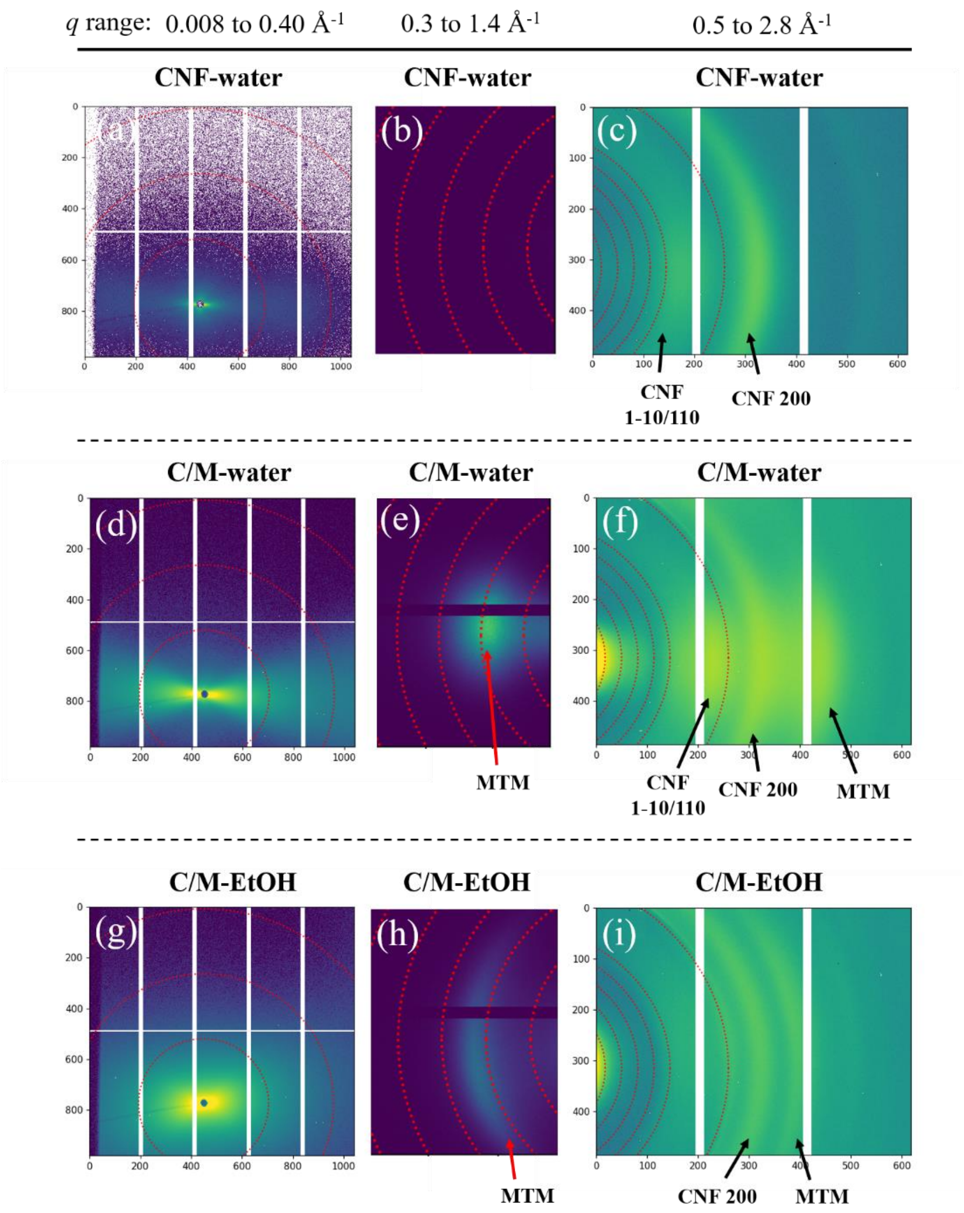

Figure S5. Examples of 2D X-ray patterns: (a, d, g) in the q region from 0.008 to 0.40 Å^{-1} (SAXS); (b, e, h) q region from 0.3 to 1.4 Å^{-1} and (c, f, i) q region from 0.5 to 2.8 Å^{-1} (WAXS) of CNF-water, C/M-water and C/M-EtOH samples. The X-ray beam is parallel to the wet mat surface.

Explanation of 2D X-ray patterns in Figure S5

Within a thin film, CNF fibrils exhibit planar orientation while MTM platelets follow uniplanar orientation.[4] Herein, anisotropic X-ray patterns are observed. After drying, CNF-water sample shows tiny SAXS streaks (Figure S5a) perpendicular to the film plane, while the composite samples (Figure S5d and S5g) show more apparent SAXS streaks, corresponding to the larger density of the film with the addition of MTM. Note that compared to C/M-EtOH, C/M-water

sample shows thinner SAXS streaks indicating a denser structure. In WAXS patterns (Figure S5c and S5f), arcing Debye rings of CNF (1-10/110) and (200) and MTM (00l) are observed. The C/M-EtOH sample (Figure S5i) show less orientated but sharper Debye rings, we discussed the possible reason in the main text.

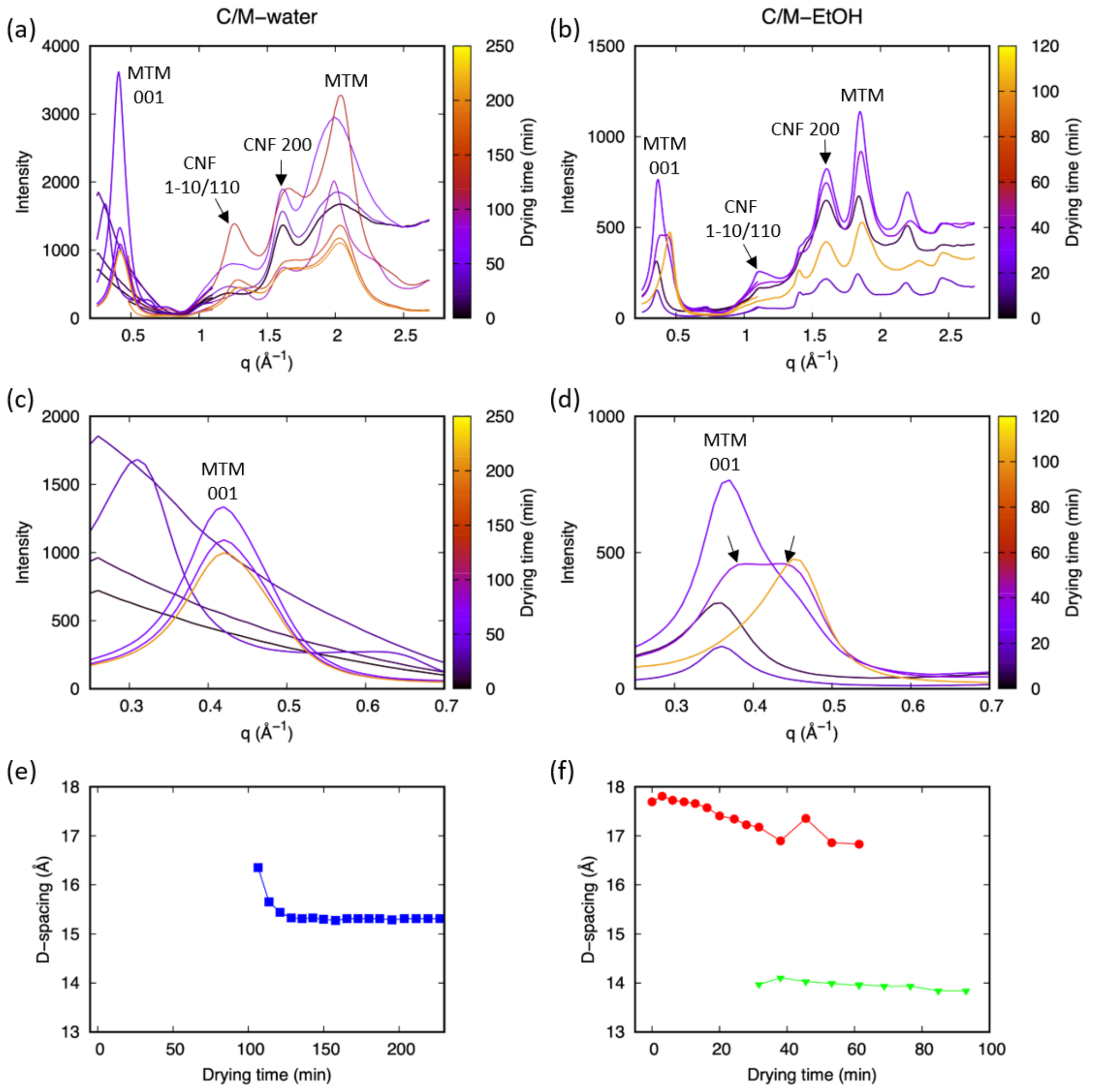

Figure S6. WAXS curves (Intensity *vs.* q) during drying: (a) C/M-water and (b) C/M-EtOH. Specific enlarged MTM 001 peaks (q near 0.3~0.5 Å^{-1}) of (c) C/M-water and (d) C/M-EtOH, the color bar indicates the drying time. D-spacing values of (e) C/M-water and (f) C/M-EtOH calculated from the MTM 001 peak based on the equation $D = \frac{2\pi}{q}$. The WAXS curves were obtained by line trace of the 2D data.

Explanation of 1D WAXS curves in Figure S6

In Figure S6a-b, we labelled the MTM and CNF peaks. The peaks in the low q region (0.3-0.5 Å^{-1}) is MTM 001 reflection, which can be used to calculate the inter-sheet distance of the stacked platelets. In Figure S6c and S6e, one can observe after drying for 100 min, the MTM 001 peaks occurred, suggesting tactoid formation; this matches well with the results in Figure

$2c_2$ in the main text. With further drying, the peaks shifted to higher q and then were relatively stable, corresponding to decreased and subsequent constant d-spacing values.

In C/M-EtOH sample, tactoids formed after solvent exchange from water to ethanol. The d-spacing of MTM in ethanol (Figure S6f, red line) is larger than that in water (18-17 Å *vs.* 16-15 Å), this is because ethanol has lower dielectric constant, dipole moment and surface tension compared to water. Ethanol penetrates the interlayer space of the MTM platelets more easily than water.[5] With further drying, the d-spacing was reduced ~ 1 Å. At about 40-50 min, two peaks were observed (indicated by the arrows in Figure S6d) corresponding to two d-spacing values, indicate the starting of moisture-induced swelling (green line) during the evaporation of ethanol. After drying for about 60 min, the ethanol was completely evaporated, and only one WAXS peak is observed.

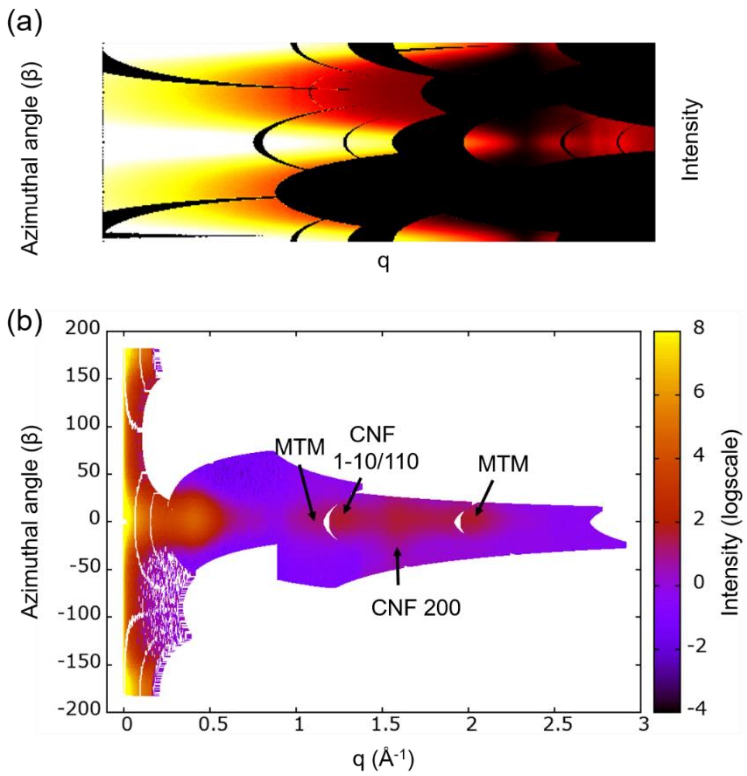

Figure S7. Intensity as a function of scattering vector q and azimuthal angle (β) covered by three detectors. Image a is an intermediate pattern to show continuous q-regions covered by three detectors, image b is a further reduced final pattern.

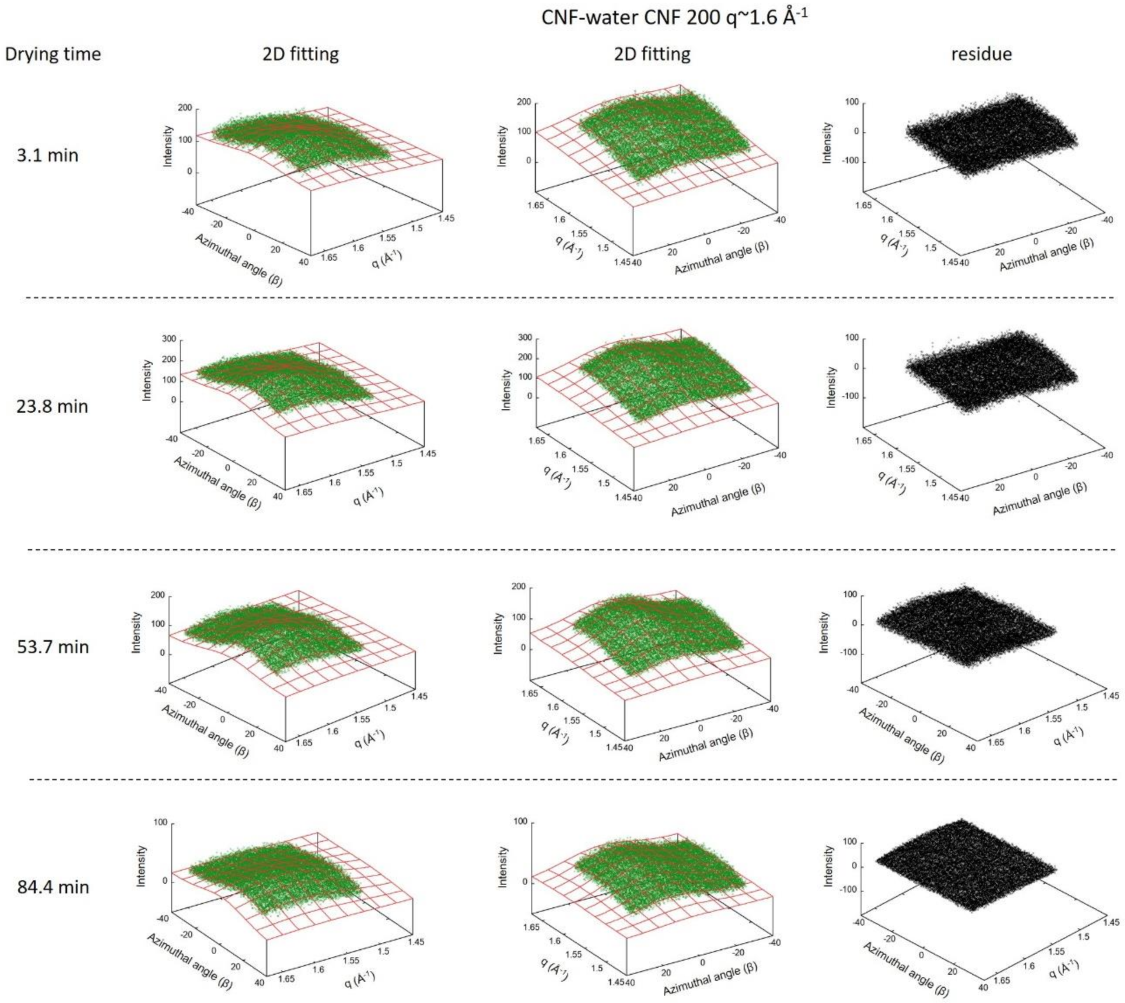

Figure S8. 2D fitting examples of CNF 200 pattern (*q* region close to 1.6 Å^{-1}) in CNF-water sample. The 1st and 2nd columns are the figures observed from different directions. The green dots are the data from experiment data, while the red mesh is from 2D Gaussian equation as described in the Experimental Section in the main text. The 3rd column is the residue after subtracting the fitted data from experimental data.

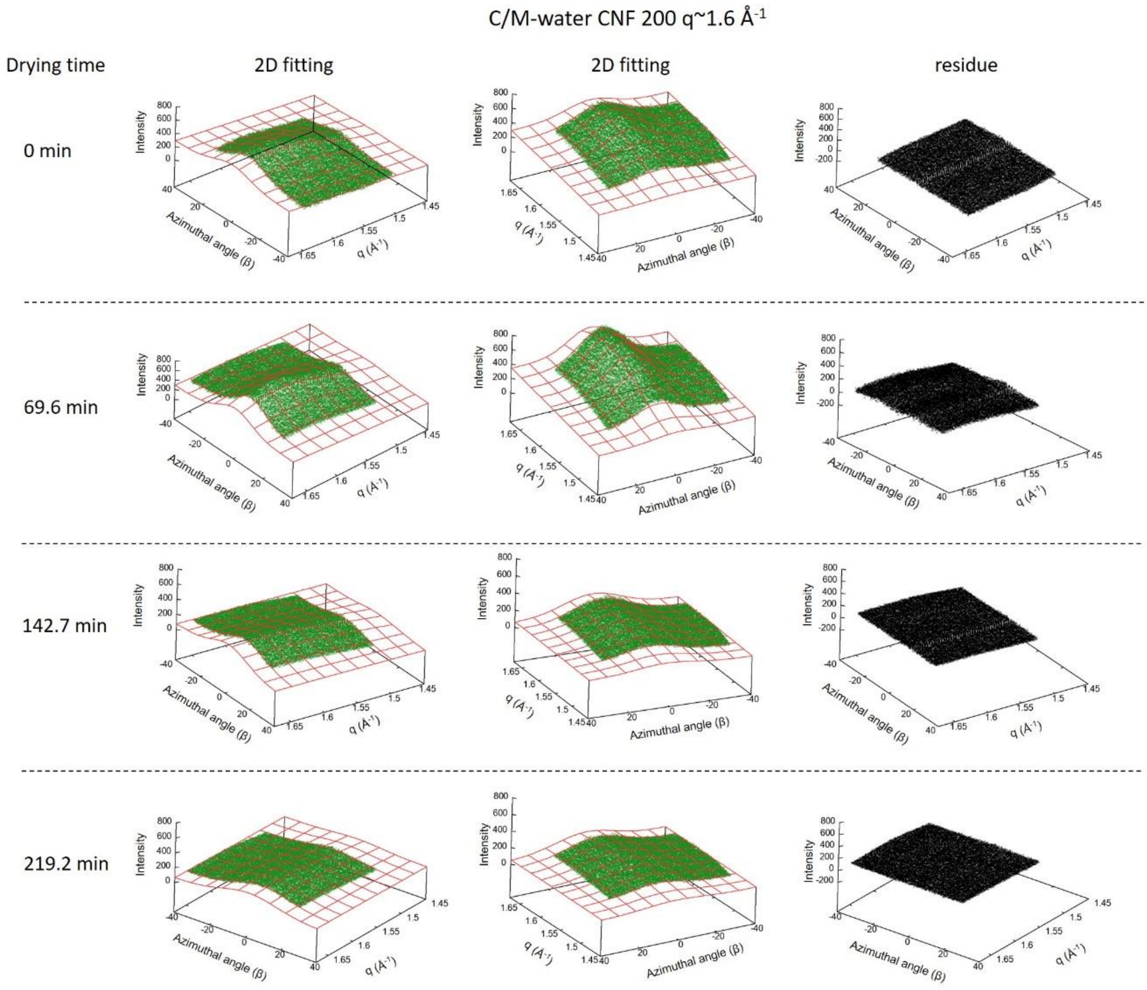

Figure S9. 2D fitting examples of CNF 200 pattern (q region close to 1.6 Å^{-1}) in C/M-water sample. The 1st and 2nd columns are the figures observed from different directions. The green dots are the data from experiment data, while the red mesh is from 2D Gaussian equation as described in the Experimental Section in the main text. The 3rd column is the residue after subtracting the fitted data from experimental data.

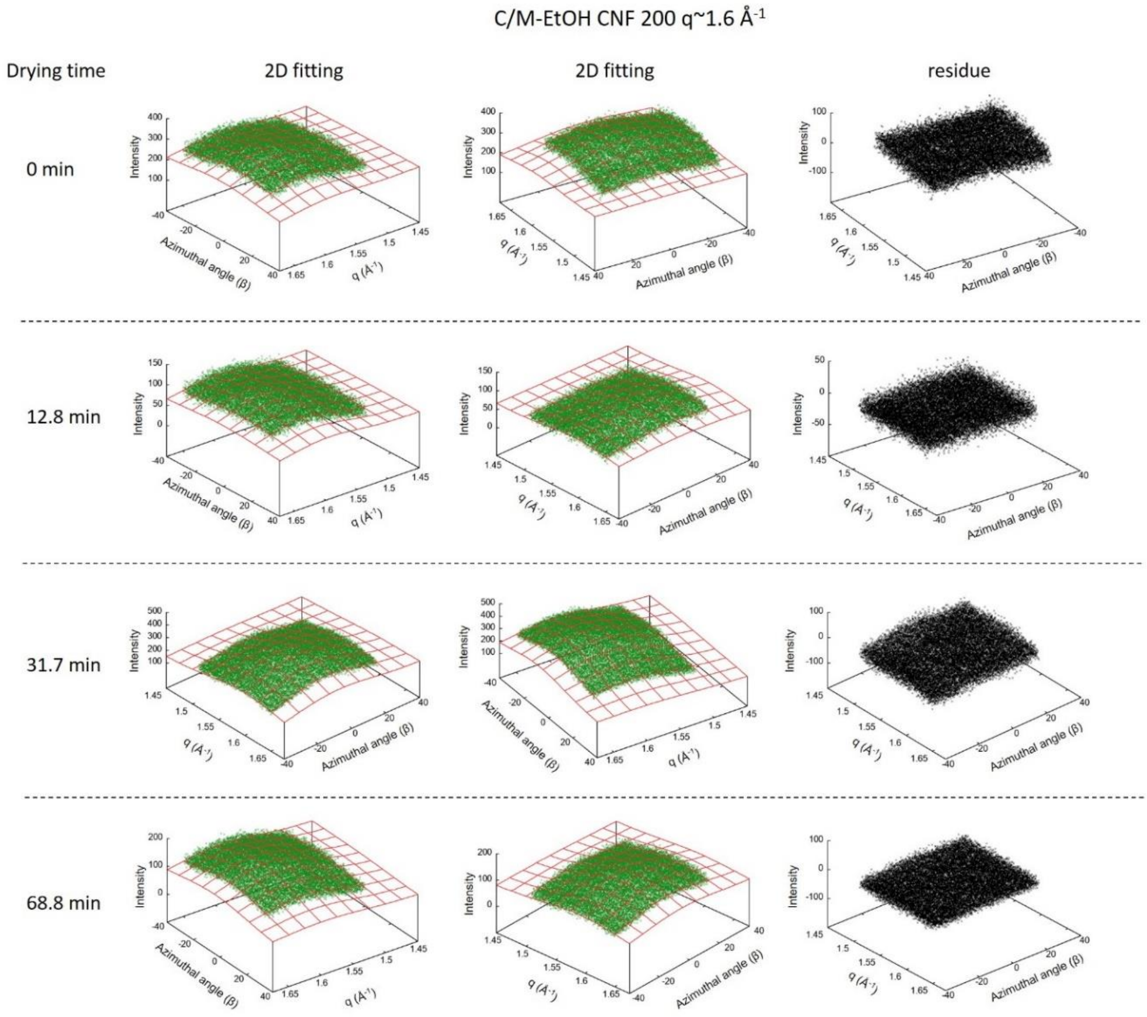

Figure S10. 2D fitting examples of CNF 200 pattern (q region close to 1.6 $Å^{-1}$) in C/M-EtOH sample. The 1st and 2nd columns are the figures observed from different directions. The green dots are the data from experiment data, while the red mesh is from 2D Gaussian equation as described in the Experimental Section in the main text. The 3rd column is the residue after subtracting the fitted data from experimental data.

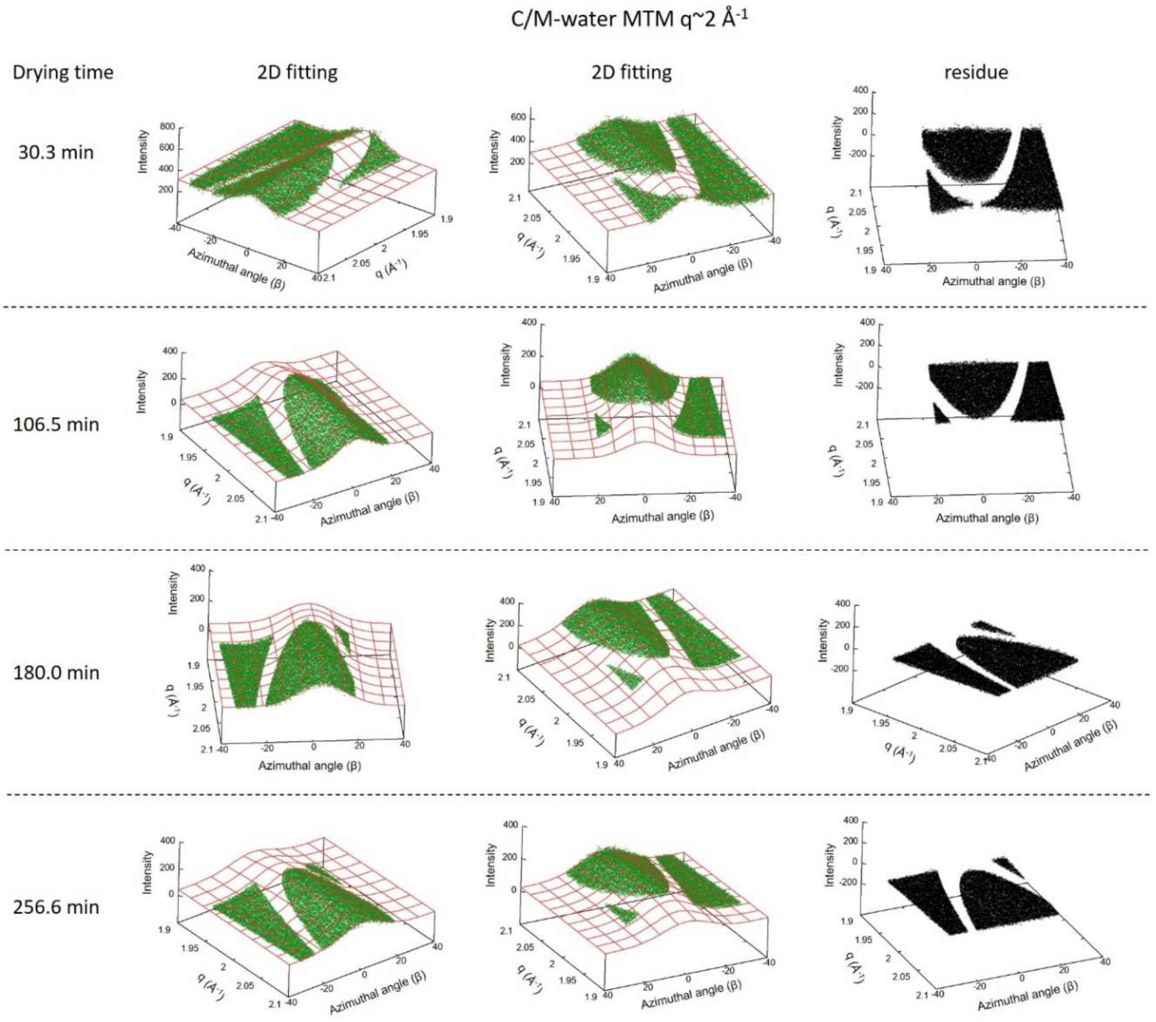

Figure S11. 2D fitting examples of MTM pattern (q region close to 2 Å^{-1}) in C/M-water sample. The 1st and 2nd columns are the figures observed from different directions. The green dots are the data from experiment data, while the red mesh is from 2D Gaussian equation as described in the Experimental Section in the main text. The 3rd column is the residue after subtracting the fitted data from experimental data.

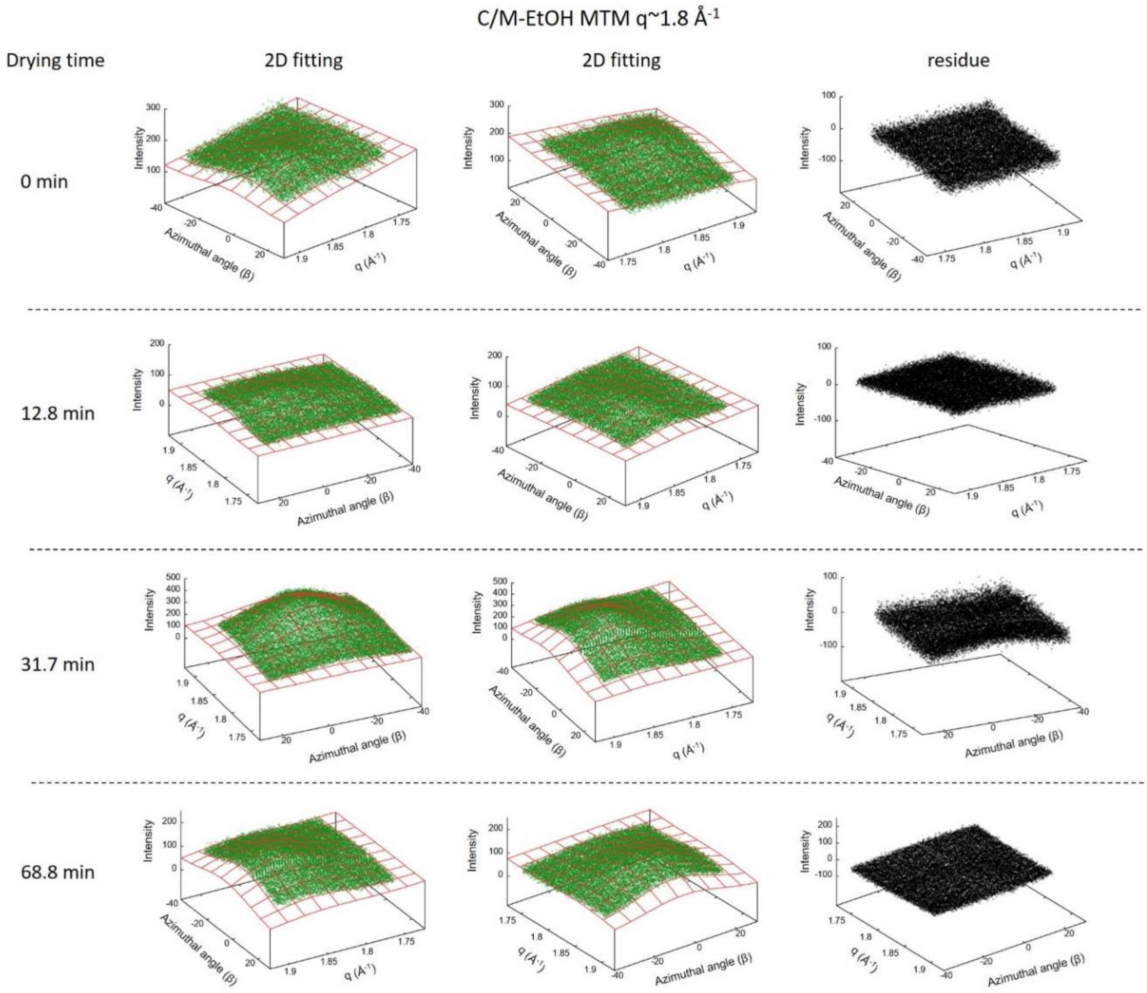

Figure S12. 2D fitting examples of MTM pattern (q region close to 2 Å^{-1}) in C/M-EtOH sample. The 1st and 2nd columns are the figures observed from different directions. The green dots are the data from experiment data, while the red mesh is from 2D Gaussian equation as described in the Experimental Section in the main text. The 3rd column is the residue after subtracting the fitted data from experimental data.

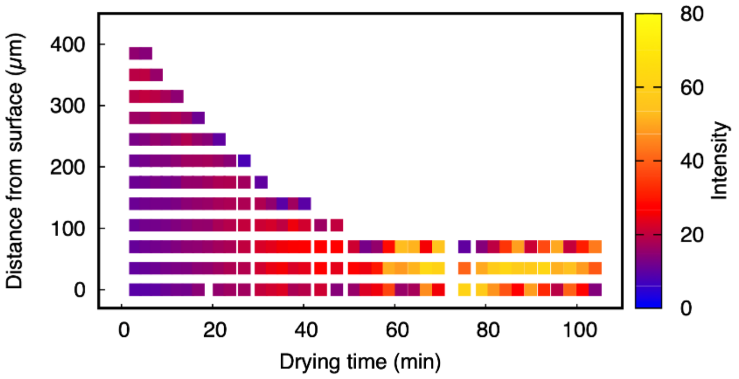

Figure S13. Palette image of peak intensity values integrated from CNF 200 pattern in CNF-water sample. The horizontal axis is drying time while the vertical axis is the distance of X-ray beam center to one reference surface of the wet mats, the color bar represents the intensity values. The standard-deviations of the parameters are smaller than 1%.

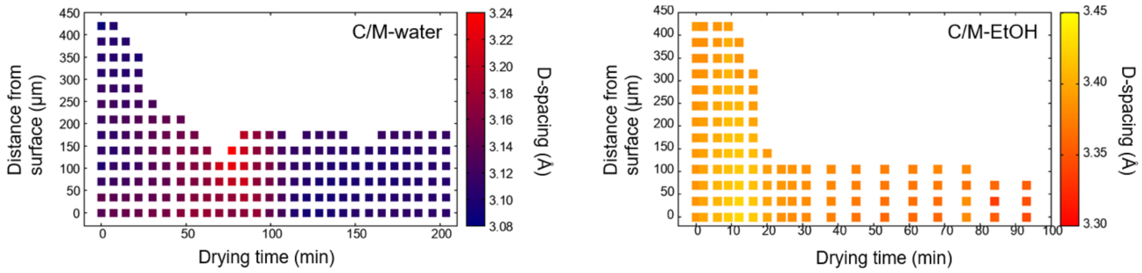

Figure S14. Palette images of MTM lateral d-spacing values extracted from MTM (q ~ 2 $Å^{-1}$) pattern in C/M-water and C/M-EtOH samples. In each image, the horizontal axis is drying time while the vertical axis is the distance of X-ray beam center to one reference surface of the wet mats, the color bar represents the values of each parameter. The standard-deviations of the parameters are smaller than 1%.

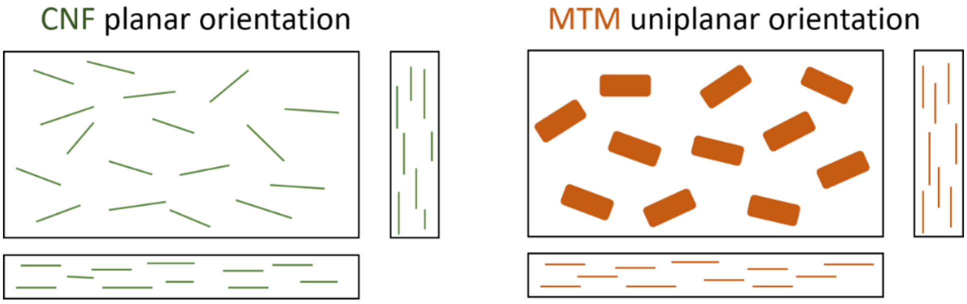

Figure S15. Illustration of CNF planar orientation and MTM uniplanar orientation. The chain axis of CNF (axial fibril direction) is parallel to the film surface and randomly distributed in the plane. The MTM platelet plane is parallel to the film plane.

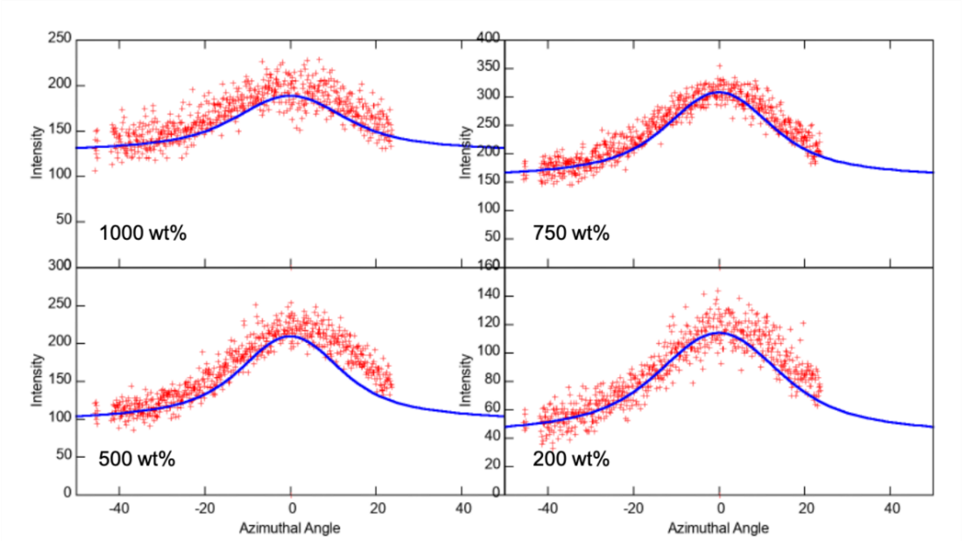

Figure S16. CNF profiles: Intensity as a function of Azimuthal angle in different water content conditions (1000, 750, 500, 200 wt %). The red dots are the experimental data of CNF 200 peak (q range 1.6 -1.605 Å^{-1}) from CNF-water sample, the blue lines are the calculated CNF profile for C/M-water sample based on the modeling method.[6]

Explanation of the artefact of CNF orientation

The CNF 200 peaks become sharper due to the overlapping of MTM peaks, so the fitted CNF orientation index is an artefact. In this case, we should analyze the realistic orientation based on a theoretical estimation: First, we obtained MTM orientation index based on the fitting of MTM peak q ~ 2.0 Å^{-1} (Figure 5b$_2$). The CNF has a fibril feature which follows the planar orientation while the MTM has a platelet feature following the uniplanar orientation (Figure S15),[4] theoretically their orientation index should be the same. By utilizing the MTM orientation index, the CNF profile along the azimuthal angle direction can be simulated by a

modeling method developed previously.[6] Figure S16 shows the simulation results, the simulated CNF profiles match well with the experimentally measured CNF profiles of CNF-water sample. This means the CNF orientation index in C/M-water is likely to be the same as for the CNF-water sample; the presence of MTM is not affecting CNF orientation.

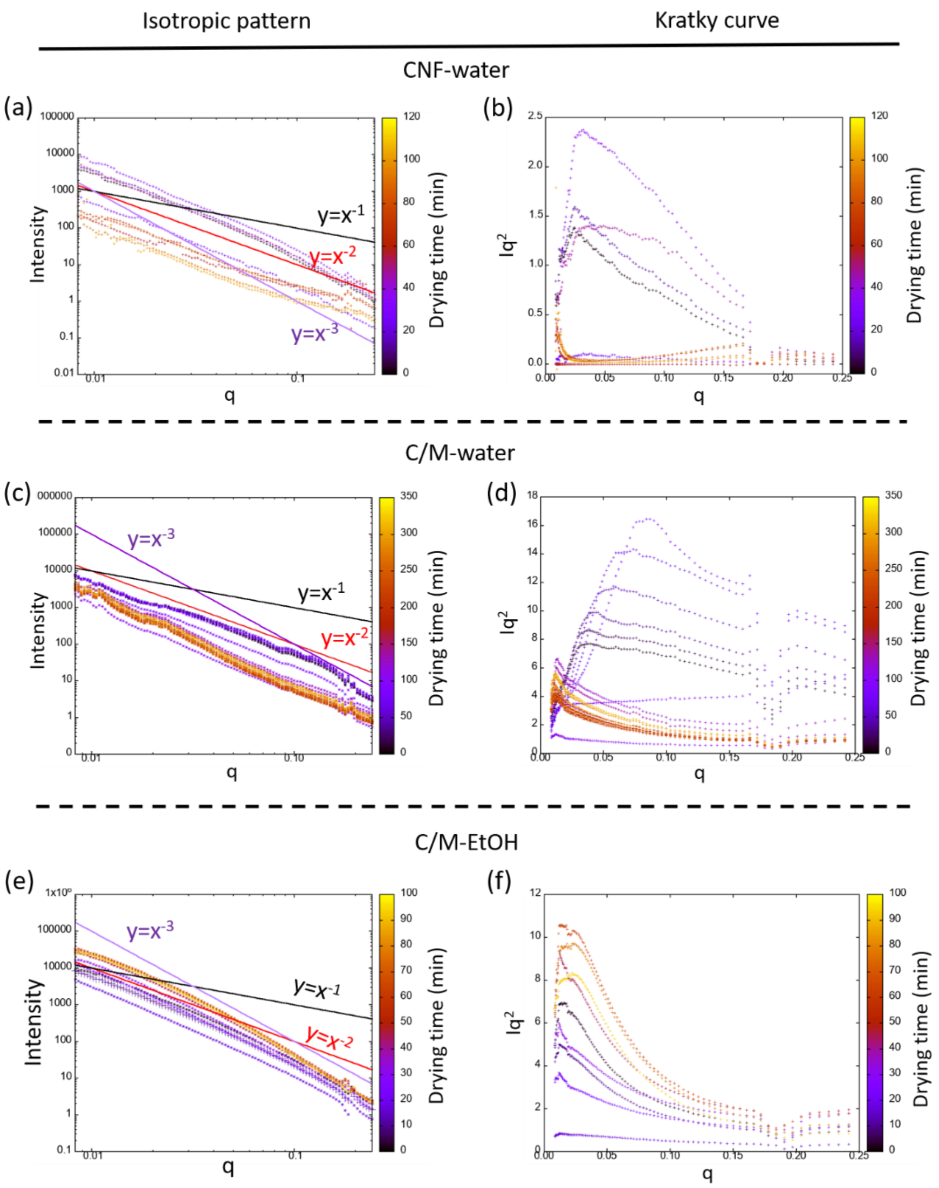

Figure S17. Extracted Isotropic SAXS curves (a, c, e) and Kratky curves (b, d, f) of CNF-water, C/M-water and C/M-EtOH samples obtained from small angle range. X-ray beam is parallel to the wet mat surface.

Surface roughness characterization

The 3D topographic features of membrane surfaces were examined by an optical surface metrology confocal profilometer (Leica DCM8, Wetzlar, Germany). Typically, samples are attached to a coverslip with adhesive tapes and placed on the moving stage. The images were captured using a lens with 20×/100× magnification and images with minimal defects and asperities were chosen for further calculating.

The roughness parameters are valid for a rectangular sampling area (A) with the lateral directions x and y and vertical direction z.[7] The arithmetical mean height of surface topography (Sa) is defined as the difference in height of each point compared to the arithmetical mean of the surface.

$$\mathrm{Sa} = \frac{1}{A}\iint_A |Z(x,y)|dxdy \tag{5}$$

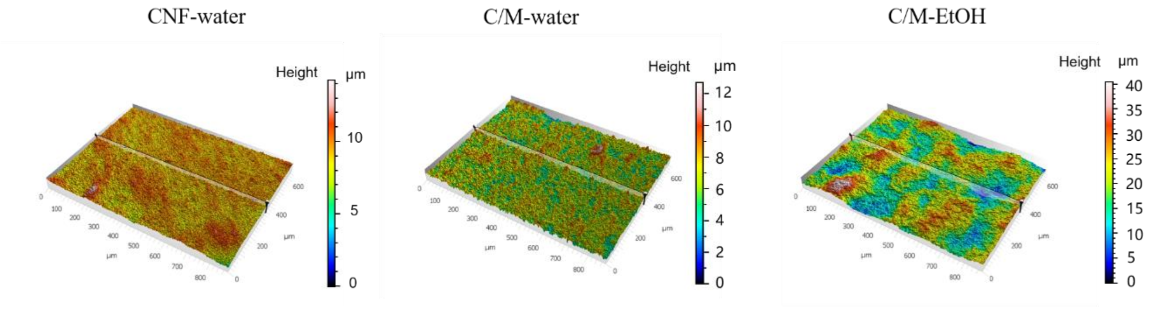

Figure S18. Topographic features of membrane surfaces after drying. C/M-EtOH shows more roughness surface.

Tensile testing method

The CNF/MTM nanocomposites films with a thickness around 30 μm were cut into a rectangle with 70 mm in length and 5 mm in width. The samples were cut by a LEICA Microtome blade after preconditioned in a 50 ± 2% relative humidity and 22 ± 1 °C room for at least 2 days. The films were tested by a Universal Testing Machine (Instron 5944, USA) equipped with a 500 N load cell and a video extensometer. The span length was set to 25 mm and the strain rate was 10% min^{-1}. All materials were tested using at least 5 specimens. The modulus was determined by fitting a linear curve from the initial elastic region.

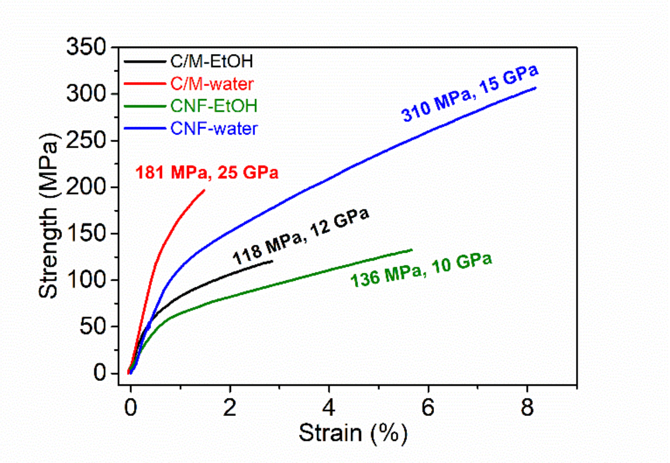

Figure S19. Representative stress-strain curves of CNF and CNF/MTM samples drying from water and ethanol.

Table S2. Mechanical property parameters of CNF and CNF/MTM samples drying from water and ethanol. In the composites, the CNF and MTM have a dry content ratio of 50/50 wt %.

**Samples**	**Modulus (GPa)**	**Ultimate strength (MPa)**	**Strain at failure (%)**	**Density (g/cm^3)**	**Porosity (%)**
CNF-water	15.5 ± 0.5	310 ± 6	7.7 ± 0.6	1.55	3.1
CNF-EtOH	9.6 ± 0.8	136 ± 8	5.4 ± 0.9	/	/
C/M-water	25.0 ± 15.4	181 ± 16	1.3 ± 0.2	1.81	12.1
C/M-EtOH	11.8 ± 1.8	118 ± 4	2.7 ± 0.3	1.36	34.0

References:

1. Evaporation from a Water Surface. *https://www.engineeringtoolbox.com/evaporation-water-surface-d_690.html*.

2. Kretschmer, C. B.; Wiebe, R., Liquid-Vapor Equilibrium of Ethanol--Toluene Solutions. *J. Am. Chem. Soc.* **1949,** *71* (5), 1793-1797.

3. Moist Air - Water Vapor and Saturation Pressure. *https://www.engineeringtoolbox.com/water-vapor-saturation-pressure-air-d_689.html*.

4. Heffelfinger, C. J.; Burton, R. L., X-Ray determination of the crystallite orientation distributions of polyethylene terephthalate films. *J. Polym. Sci.* **1960,** *47* (149), 289-306.

5. Gates, W. P., Crystalline swelling of organo-modified clays in ethanol–water solutions. *Appl. Clay Sci.* **2004,** *27* (1), 1-12.

6. Nishiyama, Y.; Johnson, G. P.; French, A. D., Diffraction from nonperiodic models of cellulose crystals. *Cellulose* **2012,** *19* (2), 319-336.

7. Järnström, J.; Ihalainen, P.; Backfolk, K.; Peltonen, J., Roughness of pigment coatings and its influence on gloss. *Appl. Surf. Sci.* **2008,** *254* (18), 5741-5749.
